# Reduction of multiscale stochastic biochemical reaction networks using exact moment derivation

**DOI:** 10.1371/journal.pcbi.1005571

**Published:** 2017-06-05

**Authors:** Jae Kyoung Kim, Eduardo D. Sontag

**Affiliations:** 1 Department of Mathematical Sciences, Korea Advanced Institute of Science and Technology, Daejeon, Korea; 2 Department of Mathematics and Center for Quantitative Biology, Rutgers University, New Brunswick, New Jersey, United States of America; Duke University, UNITED STATES

## Abstract

Biochemical reaction networks (BRNs) in a cell frequently consist of reactions with disparate timescales. The stochastic simulations of such multiscale BRNs are prohibitively slow due to high computational cost for the simulations of fast reactions. One way to resolve this problem uses the fact that fast species regulated by fast reactions quickly equilibrate to their stationary distribution while slow species are unlikely to be changed. Thus, on a slow timescale, fast species can be replaced by their quasi-steady state (QSS): their stationary conditional expectation values for given slow species. As the QSS are determined solely by the state of slow species, such replacement leads to a reduced model, where fast species are eliminated. However, it is challenging to derive the QSS in the presence of nonlinear reactions. While various approximation schemes for the QSS have been developed, they often lead to considerable errors. Here, we propose two classes of multiscale BRNs which can be reduced by deriving an exact QSS rather than approximations. Specifically, if fast species constitute either a feedforward network or a complex balanced network, the reduced model based on the exact QSS can be derived. Such BRNs are frequently observed in a cell as the feedforward network is one of fundamental motifs of gene or protein regulatory networks. Furthermore, complex balanced networks also include various types of fast reversible bindings such as bindings between transcriptional factors and gene regulatory sites. The reduced models based on exact QSS, which can be calculated by the computational packages provided in this work, accurately approximate the slow scale dynamics of the original full model with much lower computational cost.

## Author summary

Molecules inside a cell undergo various transformations via biochemical reactions with disparate rates. For instance, while transcriptional factors bind and unbind gene promoters in a time scale of seconds, mRNA transcription takes at least several minutes. For such systems regulated by both fast and slow reactions together, most of the computation time in stochastic simulations is spent on simulating the fast reactions, even if our interest is in the dynamics of slow reactions such as transcription. This problem can be resolved by deriving a reduced system, which can accurately approximate the slow dynamics of the original system without simulating fast reactions. However, when nonlinear reactions exist, the accurate reduction is often impossible. Here, we describe that two classes of nonlinear systems, both of which appear frequently as models of natural biochemical reaction networks, can be effectively reduced, and we provide a computational package to carry out the reduction. We find that the resulting reduced systems accurately capture the stochastic dynamics of the original system, while saving considerable simulation time.

## Introduction

Many biochemical reaction networks (BRNs) in a cell include species whose copy numbers are small [1–3] (e.g. one or a few copies of genes or tens of mRNAs), which lead to large fluctuations in the system. Such stochastic dynamics of BRNs can be described by the chemical master equation (CME), but the analytic solutions of the CME cannot be calculated in most cases. Thus, to obtain numerical solutions of the CME, the Gillespie algorithm has been widely used, which generates exact sample paths of the CME [4]. However, such numerical simulation becomes extremely inefficient in the presence of stochastic stiffness [5, 6]. Specifically, in the presence of fast reactions, stochastic simulations become slow as the computation is predominantly spent on simulating fast reactions. Thus, it becomes computationally prohibitive to use the Gillespie algorithm to simulate multiscale BRNs.

One approach to resolve this problem is utilizing timescale separation among species in multiscale BRNs [7–13]. In such networks, fast species regulated by fast reactions will quickly equilibrate to their stationary distributions while slow species remain constant. Thus, on the slow timescale, the fast species in propensity functions of reactions rapidly average out to their quasi-steady-state (QSS): their stationary conditional expectation value for given states of slow species. As the QSS is solely determined by the state of slow species, replacement of the fast species with their QSS leads to a reduced model, where fast species are eliminated. This reduced system accurately captures the dynamics of slow species of the original full system on the slow timescale without simulating fast species and thus with much lower computational cost.

In the presence of nonlinear reactions, moment equations usually involve infinite ODEs and thus deriving the exact QSS becomes challenging. Thus, various approximations for QSS have been proposed [8, 14–27]. However, the validity conditions have often been tested with some toy examples and thus general validity conditions of such approximations have not been fully understood. For instance, a popular approximation is using the QSS of the corresponding deterministic system (e.g. large volume limit of the stochastic system) [8, 21–23]. Specifically, deterministically derived QSS such as Michaelis-Menten and Hill functions has been used to approximate the stochastic QSS and derive the propensity functions of the Gillespie algorithm. Despite the popularity of this approach [28–44], it can lead to considerable errors depending on the parameter choice [9, 45–51]. Furthermore, its validity conditions have been investigated only recently [45–51] and have not been fully identified yet for general cases.

Despite the difficulty in the derivation of the exact QSS in general, recent studies have revealed that such derivations are possible for several special cases of nonlinear BRNs (see [52] for a recent review for this topic). Stationary solutions of CMEs associated to enzyme kinetics have been studied in [53–55]. In particular, Levine and Hwa used the exact QSS of an enzyme kinetics system to reduce various types of models that appear in metabolic pathways [56]. Furthermore, the stationary distribution for a single gene regulatory model was also derived recently [57–59].

In this work, rather than focusing on a specific case, we investigate the exact QSS of two general classes of nonlinear BRNs, which are frequently found in many natural biological systems. The first class is BRNs whose fast subnetwork is a feedforward network. In contrast to linear systems, moment equations of nonlinear systems depend on higher orders, and lead in general to an infinite system of coupled ODEs. However, in the recent work [60], Sontag and Singh proved that all moments of a certain class of nonlinear feedforward networks can be computed by finite linear differential equations. We will describe how this theory can be used to derive an accurate reduction of a nonlinear BRN whose fast subnetwork is a feedforward network. To aid in implementations, we provide a computational package, FEEDME, which calculates the moments of interest for systems with a linear moment closure property and thus the exact QSS of the embedded feedforward network.

In [61], Anderson, Craciun, and Kurtz showed how stationary distributions of a certain broad class of BRNs, namely complex balanced networks, have the form of a multivariate Poisson distribution normalized by an appropriate partition function which accounts for conservation laws; they then applied their result to several examples, including a model of multiscale enzyme kinetics. The key difficulty in obtaining the stationary distribution and thus stationary moments becomes then to compute this partition function. In [62], Sontag and Zeilberger developed an approach, based on transforms and factorial moments, to compute this function as well as all first and higher-order moments, for complex balanced networks. Moreover, Wilf-Zeilberger theory led to a formula that computes the partition function recursively on integer conserved amounts (see Methods for details on the basic theorems from [61] and [62]). The applicability of these results for the model reduction was not studied in [62], however. Recently, Mélykúti, Hespanha, and Khammash in [63] derived analytic expressions for an extensive collection of nonlinear BRNs, including reversible bindings on path-like, circular, and ladder-shaped state spaces, also framing the discussion in the context of complex balancing, and explained how those results can be used in model reduction, but [63] did not take advantage of the methods and formulas in the paper [62] which provide a general theoretical and algorithmic approach in the complex balanced case. In the present paper, we make full use of the theory in [62] in order to effectively derive an exact QSS for complex balanced networks. This provides a systematic way to perform the time-scale reduction for the second class of BRNs, those whose fast subnetwork constitutes a complex balanced network.

We illustrate the procedure of QSS derivation, and thus reduction, for both classes of systems with several examples, including a transcriptional negative feedback loop combined with a fast feedforward signaling pathway, a genetic oscillator with a fast reversible binding and a transcriptional positive feedback loop with fast competitive reversible bindings. In every case, we show how the reduced models based on the exact QSS outperform those based on the approximate QSS.

## Results

### Reduction of a multiscale feedforward network

Feedforward networks are frequently observed in gene and protein regulatory networks as they play the critical roles in response of system for given signals [64, 65]. In the class of feedforward networks considered in this work, species can be partitioned into a series of layers so that every nonlinear reaction among multimers lead to the production of a new species in later layers without destruction of multimers (see Methods for details). Importantly, such feedforward networks have the linear moment closure property, and thus any of their moments can be calculated using Eq (15) recursively even in the presence of nonlinear reactions [60]. Our algorithm, FEEDME, uses such a property to calculate moments of interest. In particular, the stationary conditional moments of fast species given slow species can be derived, which allows for accurate reductions of multiscale stochastic systems.

**Fig 1:**
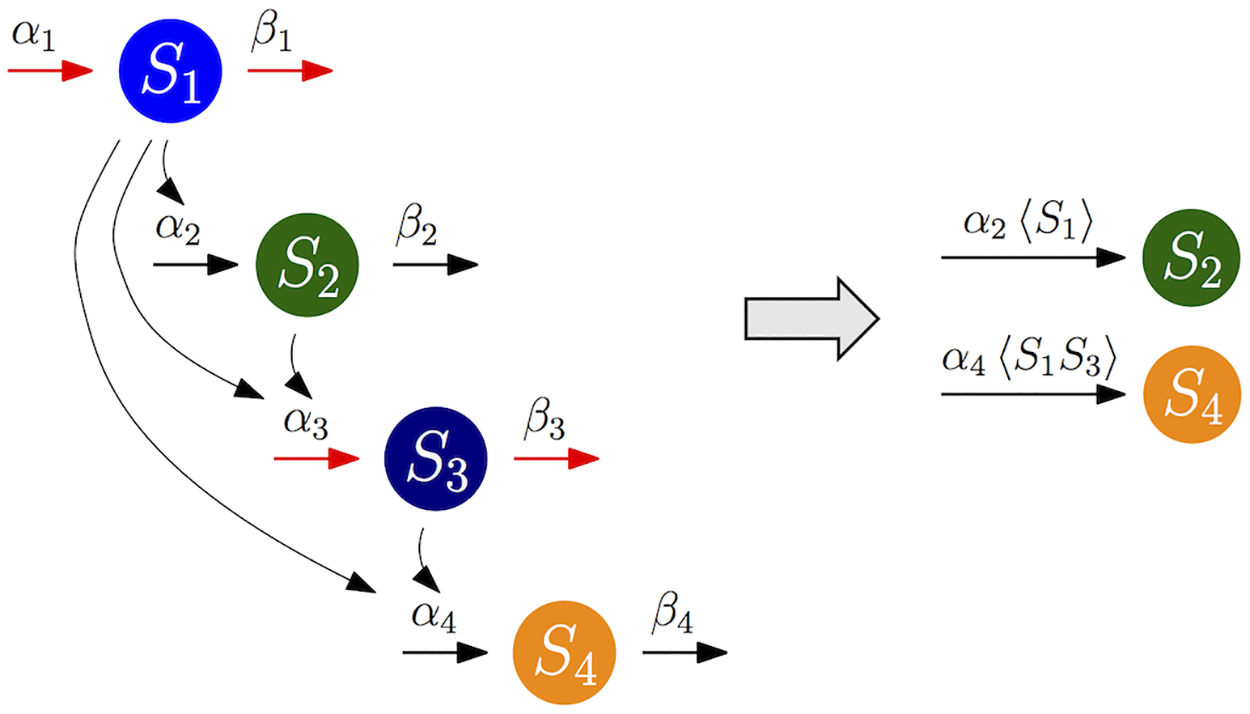
Model diagram of the feedforward network of the full model and the reduced model. In the diagram of the full model (left), red arrows indicate fast reactions. In the diagram of the reduced model (right), which consists of slow species *S*_2_ and *S*_4_ and slow reactions, 〈*S*_1_〉 and 〈*S*_1_*S*_3_〉 represent conditional moments, 〈*X*_1_|*X*_2_, *X*_4_〉 and 〈*X*_1_*X*_3_|*X*_2_, *X*_4_〉, respectively. In both of the diagrams, degradation reactions are not shown, for simplicity.

Here, we illustrate this reduction process using an example of multiscale nonlinear feedforward network (Fig 1). This system consists of 4 species and 8 reactions (Table 1): *S*_1_ is constitutively produced. *S*_1_, a heterodimer consisting of *S*_1_ and *S*_2_, and a heterodimer consisting of *S*_1_ and *S*_3_ promote the production of *S*_2_, *S*_3_, and *S*_4_, respectively. *S*_*i*_ degrades with its own rate. Propensity functions are derived based on mass action kinetics by defining *X*_*i*_(*t*) be the abundance of the species *S*_*i*_ at time *t* (Table 1).

**Table 1:** Reactions and propensity functions in the feedforward network (Fig 1).

Reactions	Propensity functions
$\overset{\alpha_{1}}{\to }S_{1}$	*α*_1_Ω
$S_{1}\overset{\beta_{1}}{\to }$	*β*_1_*X*_1_
$S_{1}\overset{\alpha_{2}}{\to }S_{1}+S_{2}$	*α*_2_*X*_1_
$S_{2}\overset{\beta_{2}}{\to }$	*β*_2_*X*_2_
$S_{1}+S_{2}\overset{\alpha_{3}}{\to }S_{1}+S_{2}+S_{3}$	$\frac{\alpha_{3}}{\Omega }X_{1}X_{2}$
$S_{3}\overset{\beta_{3}}{\to }$	*β*_3_*X*_3_
$S_{1}+S_{3}\overset{\alpha_{4}}{\to }S_{1}+S_{3}+S_{4}$	$\frac{\alpha_{4}}{\Omega }X_{1}X_{3}$
$S_{4}\overset{\beta_{4}}{\to }$	*β*_4_*X*_4_

Here, *α*_1_Ω = 2/*ϵ* *m*^−1^, *α*_3_/Ω = 2/*ϵ* *m*^−1^, *α*_2_ = 2 *m*^−1^, *α*_4_/Ω = 2 *m*^−1^, *β*_1_ = *β*_3_ = 1/*ϵ* *m*^−1^, and *β*_2_ = *β*_4_ = 1 *m*^−1^. Ω represents the volume of the system and *ϵ* ≪ 1.

As reactions involving the synthesis and degradation of *S*_1_ and *S*_3_ are faster than other reactions (Fig 1 and Table 1), *S*_1_ and *S*_3_ are fast species that rapidly fluctuate and quickly equilibrate to their stationary distribution conditioned on the slow species. Thus, the propensity functions of slow reactions involving fast species, *α*_2_*X*_1_ and (*α*_4_/Ω)*X*_1_*X*_3_, rapidly equilibrate to their stationary conditional expectation values, *α*_2_〈*X*_1_|*X*_2_, *X*_4_〉 and (*α*_4_/Ω) 〈*X*_1_*X*_3_|*X*_2_, *X*_4_〉, respectively. Our algorithm, FEEDME, allows for calculating these moments in terms of slow species:

$$\begin{array}{ccc} \alpha_{2}\langle X_{1}|X_{2},X_{4}\rangle & = & \frac{\alpha_{1}\alpha_{2}\Omega }{\beta_{1}}, \end{array}$$ (1)

$$\begin{array}{ccc} \alpha_{4}/\Omega \langle X_{1}X_{3}|X_{2},X_{4}\rangle & = & \alpha_{4}X_{2}\left(\frac{\alpha_{1}^{2}\alpha_{3}}{\beta_{1}^{2}\beta_{3}}+\frac{\alpha_{1}\alpha_{3}}{\left(\beta_{1}+\beta_{3}\right)\beta_{1}\Omega }\right). \end{array}$$ (2)

By substituting these QSS for the original propensity functions, we can derive a reduced system, which solely consists of slow species *S*_2_ and *S*_4_ (Fig 1). We refer to this reduced model as the EMB model (exact-moment based model) for this example. When such exact moments cannot be calculated, an alternative popular approach uses the deterministically derived QSS under the moment closure assumption (〈*X*_*i*_*X*_*j*_〉 = 〈*X*_*i*_〉〈*X*_*j*_〉) [8, 21–23] as follows:

$$\begin{array}{c} \alpha_{4}/\Omega_{1}\langle X_{1}X_{3}|X_{2},X_{4}\rangle \approx \alpha_{4}/\Omega \langle X_{1}|X_{2},X_{4}\rangle \langle X_{3}|X_{2},X_{4}\rangle =\alpha_{4}X_{2}\left(\frac{\alpha_{1}^{2}\alpha_{3}}{\beta_{1}^{2}\beta_{3}}\right). \end{array}$$ (3)

Note that this approximate moment is the limit of the exact moment Eq (2) as Ω → ∞ (i.e. as one gets closer to the deterministic system). We refer to the reduced model based on this approximate moment as the AMB model (approximate-moment based model) for this example.

**Fig 2:**
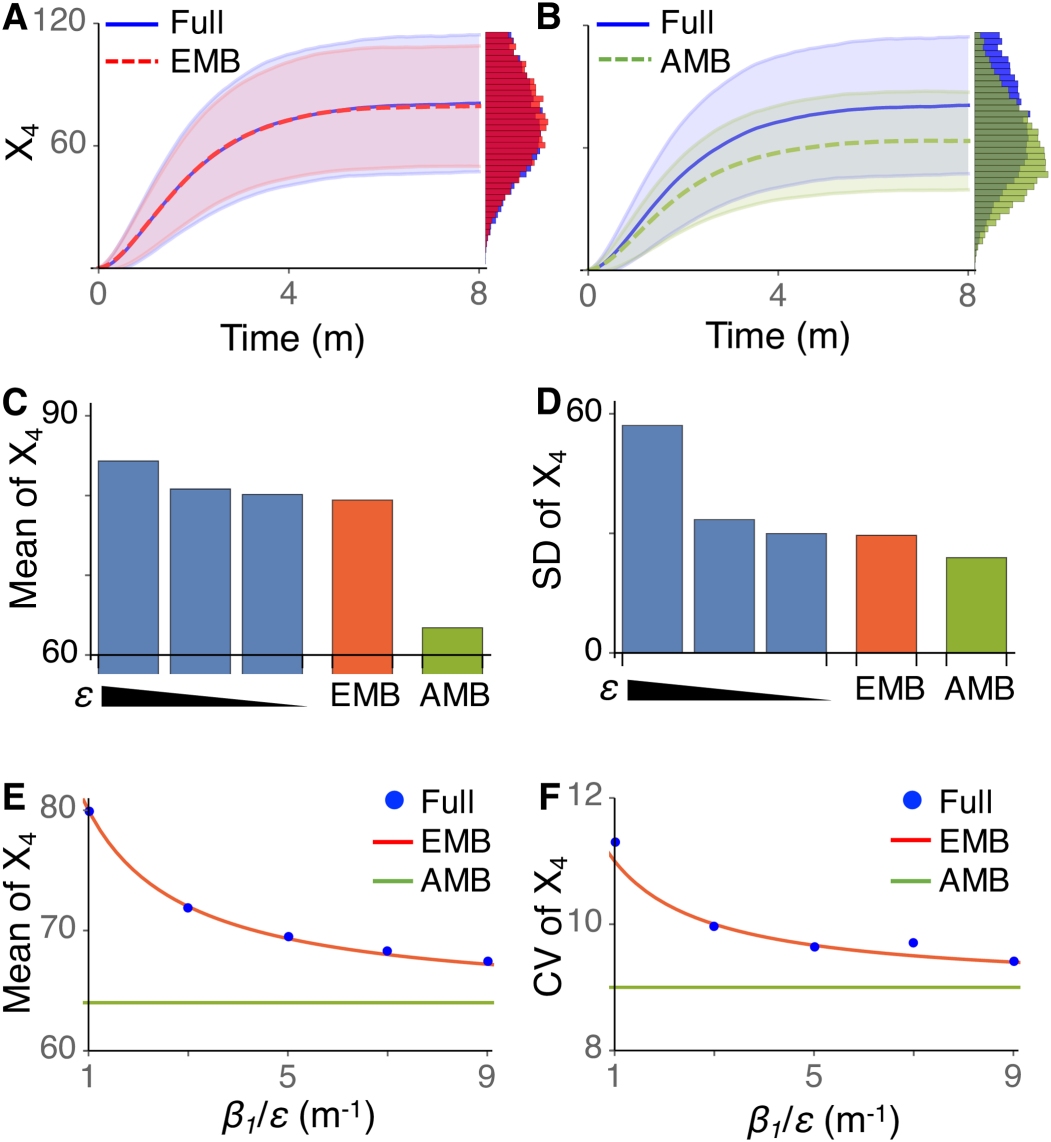
EMB model provides much more accurate approximation of the original feedforward network model than AMB model. (A-B) Trajectories of original full model with *ϵ* = 0.01 and the EMB model (A) and the AMB model (B). The lines and colored ranges indicate the mean of *X*_4_ and standard deviations of *X*_4_ from their mean, respectively. Histograms represent distributions of *X*_4_ at the steady state. Here, *X*_1_(0) = *α*_1_/*β*_1_, *X*_2_(0) = *X*_3_(0) = *X*_4_(0) = 0. Here, 10^4^ stochastic simulations were performed. (C-D) Mean (C) and standard deviation (D) of stationary distribution (t = 8) simulated with the full model with varying *ϵ* = 0.1, 0.05, 0.01, the EMB model and the AMB model. (E-F) As *β*_1_ = *α*_1_ increases, the EMB model, but not the AMB model, predicts that the mean and the CV of *S*_4_ decrease, which is consistent with the simulations of the original full model.

We compared the stochastic simulations of the full model with *ϵ* = 0.01, the EMB model and the AMB model. The mean and standard deviation of simulated trajectories of the full model is accurately captured by the EMB model (Fig 2A), but not by the AMB model (Fig 2B). In addition, as *ϵ* decreases, both the mean and the standard deviation of *S*_4_ at the steady state of the full model approach those of the EMB model (Fig 2C), but not those of the AMB model (Fig 2D). This indicates that the stochastic reduction based on exact moments is accurate as long as there is a large enough timescale separation. This is consistent with previous theoretical studies [8, 26]. Furthermore, note that as *ϵ* decreases, not only does the accuracy of the EMB model increase, but also a longer computation time would be required for the simulation of the full model, rendering even more clear the benefit of our reduction. Taken together, these results provide evidence that our algorithm, FEEDME, allows for accurate stochastic reductions for mutli-scale feedforward networks.

Next, we illustrate how a key property of the original full model can be revealed only by the EMB model, but not by the AMB model. In the feedforward network that we study, a critical issue is understanding the relationship between input molecules (*S*_1_) and downstream output molecules (*S*_4_). Suppose that the production rate (*α*_1_) and the degradation rate (*β*_1_) of *S*_1_ change proportionally, which ensures that the average copy number of *S*_1_ is invariant. A natural question is: do the average copy number and the fluctuation level of *S*_4_ change? From Eq (3), the stationary average number of *S*_4_ in the AMB model can be derived as follows:

$$\begin{array}{c} \langle X_{4}\rangle =\frac{\alpha_{4}}{\beta_{4}}\langle X_{2}\rangle \left(\frac{\alpha_{1}^{2}\alpha_{3}}{\beta_{1}^{2}\beta_{3}}\right)=\Omega {\left(\frac{\alpha_{1}}{\beta_{1}}\right)}^{3}\frac{\alpha_{2}\alpha_{3}\alpha_{4}}{\beta_{2}\beta_{3}\beta_{4}}, \end{array}$$ (4)

which predicts that 〈*X*_4_〉 does not change as long as $\frac{\alpha_{1}}{\beta_{1}}$ and thus $\langle X_{\text{1}}\rangle =\Omega \frac{\alpha_{1}}{\beta_{1}}$ are maintained. However, the stationary average number of *S*_4_ derived with the EMB model using Eq (2) leads to a different conclusion, namely:

$$\begin{array}{c} \langle X_{4}\rangle =\frac{\alpha_{4}}{\beta_{4}}\langle X_{2}\rangle \left(\frac{\alpha_{1}^{2}\alpha_{3}}{\beta_{1}^{2}\beta_{3}}+\frac{\alpha_{1}\alpha_{3}}{\left(\beta_{1}+\beta_{3}\right)\beta_{1}\Omega }\right)=\Omega {\left(\frac{\alpha_{1}}{\beta_{1}}\right)}^{3}\frac{\alpha_{2}\alpha_{3}\alpha_{4}}{\beta_{2}\beta_{3}\beta_{4}}+{\left(\frac{\alpha_{1}}{\beta_{1}}\right)}^{2}\frac{\alpha_{2}\alpha_{4}}{\beta_{2}\beta_{4}}\frac{\alpha_{3}}{\left(\beta_{1}+\beta_{3}\right)}, \end{array}$$ (5)

which decreases even when *α*_1_ and *β*_1_ increase proportionally. This is consistent with the simulation of the full model (Fig 2E). Furthermore, as, in contrast to the original full model, the AMB and the EMB model are linear, the coefficient of variation of *S*_4_ in both the AMB and the EMB model can be easily derived, and it is

$$\begin{array}{c} 1+\frac{\langle X_{4}\rangle }{\langle X_{2}\rangle }\frac{1}{1+\beta_{2}/\beta_{4}}. \end{array}$$

In the AMB model, 〈*X*_4_〉 does not change, as long as 〈*X*_1_〉 is maintained constant, and thus the C.V. of *S*_4_ remains constant. On the other hand, the C.V. of *S*_4_ in the EMB model decreases even when 〈*X*_1_〉 is maintained constant due to a proportional increase in its production and degradation rate, a behavior which is also consistent with the full model (Fig 2F). Taken together, these two observations show that EMB model, but not the AMB model, can accurately capture an important feature of the original full model, namely that average copy number and fluctuation level of the output molecule (e.g. *S*_4_) in the feedforward loop depends both on the timescale of the input molecule (i.e. *S*_1_) and on its average level. This illustrates the importance of the accurate model reduction and its advantage over the original full model.

### Reduction of a multiscale system with an embedded fast feedforward subnetwork

Previously, we illustrated how our algorithm, FEEDME can be used to derive the accurate reduction of the multiscale feedforward network (Fig 1). In fact, FEEDME can be used to obtain an accurate reduction of more general BRNs. For the reduction, calculation of all the moments is not necessary, and knowing the conditional moments of certain fast species involved with the slow propensity functions is enough. Thus, as long as the fast subnetwork of the BRN is a feedforward network, the conditional moments of the fast species can be derived with the FEEDME algorithm, and thus an accurate reduction is possible.

**Fig 3:**
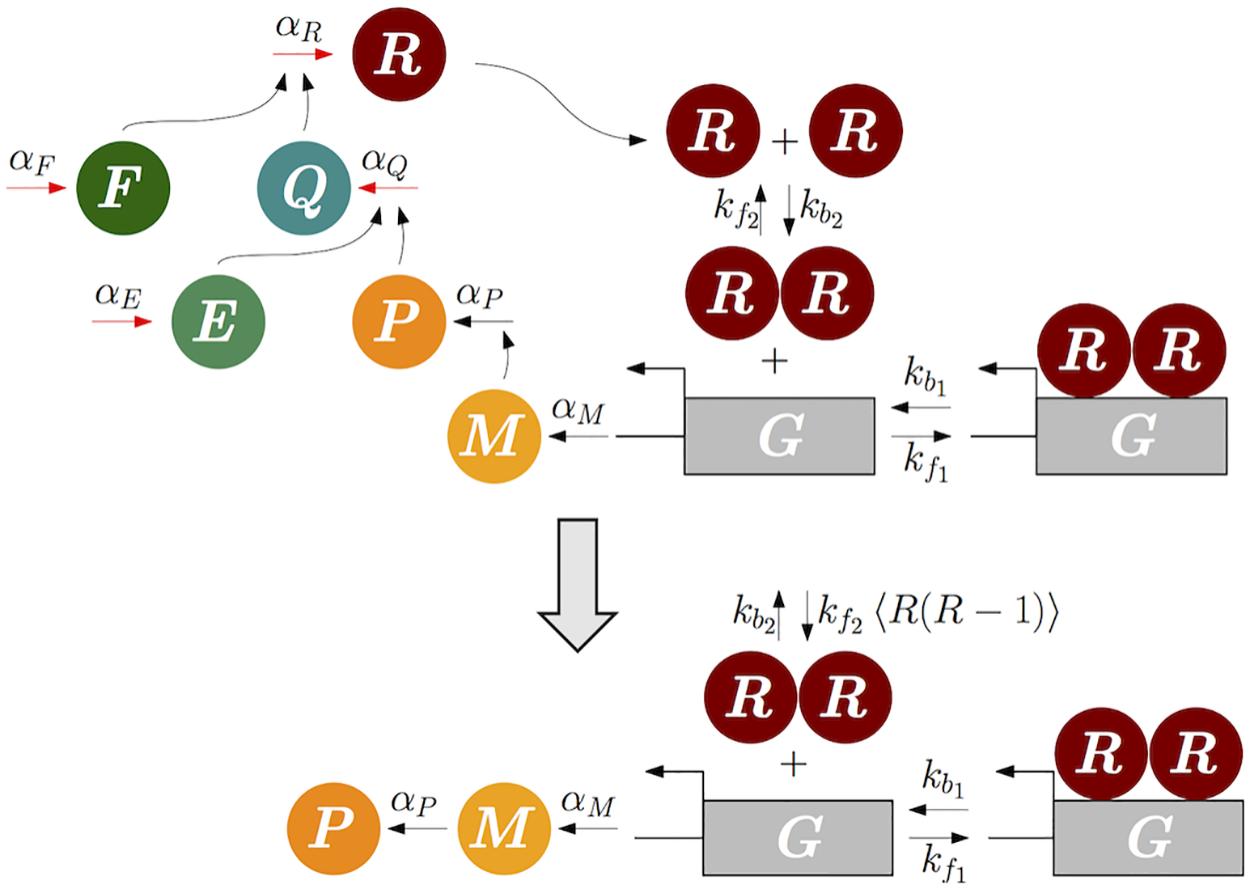
Model diagram of a transcriptional negative feedback loop with a fast feedfoward subnetwork and a slow dimerization. In the diagram of the full model (top), red arrows indicate fast reactions. Note that the subnetwork consisting of the fast species *E*, *Q*, *F*, and *R* with fast reactions (red arrows) is feedforward. In the diagram of the reduced model (bottom), which solely consists of slow species and slow reactions, 〈*R*(*R* − 1)〉 represents a conditional moment, 〈*X*_*R*_(*X*_*R*_ − 1)|*X*_*P*_〉. In both of the diagrams, all degradation reactions are not shown, for simplicity.

We illustrate this case using an example of a transcriptional negative feedback loop with a dimerization (Fig 3). This system consists of 16 reactions including fast feedforward reactions and slow reversible bindings (Table 2): active gene (*G*) promotes the transcription of mRNA (*M*), which is then translated to the protein (*P*). This protein acts as an enzyme which triggers the production of repressor (*R*) via a fast feedforward network, which involves fast species, *Q*, *E* and *F*. Specifically, *E* functions as a cofactor for the protein *P* to be able to promote the production of *Q*. Similarly, *F* also functions as a cofactor for the protein *Q* so that *Q* can promote the production of repressor protein *R*. Then, dimerized *R*: *R* reversibly binds with *G* to form repressed DNA complex (*G*_*R*_).

**Table 2:** Reactions and propensity functions in the transcriptional negative feedback loop with a dimerization (Fig 3).

Reactions	Propensity functions
$G+R:R\overset{k_{f_{1}}}{\to }G_{R}$	$\frac{k_{f_{1}}}{\Omega }X_{G}X_{R:R}$
$G_{R}\overset{k_{b_{1}}}{\to }G+R:R$	$k_{b_{\text{1}}}X_{G_{R}}$
$G\overset{\alpha_{M}}{\to }G+M$	*α*_*M*_*X*_*G*_
$M\overset{\beta_{M}}{\to }$	*β*_*M*_*X*_*M*_
$M\overset{\alpha_{P}}{\to }M+P$	*α*_*P*_*X*_*M*_
$P\overset{\beta_{P}}{\to }$	*β*_*P*_*X*_*P*_
$\overset{\alpha_{E}}{\to }E$	*α*_*E*_Ω
$E\overset{\beta_{E}}{\to }$	*β*_*E*_*X*_*E*_
$E+P\overset{\alpha_{Q}}{\to }E+P+Q$	$\frac{\alpha_{Q}}{\Omega }X_{E}X_{P}$
$Q\overset{\beta_{Q}}{\to }$	*β*_*Q*_*X*_*Q*_
$\overset{\alpha_{F}}{\to }F$	*α*_*F*_Ω
$F\overset{\beta_{F}}{\to }$	*β*_*F*_*X*_*F*_
$Q+F\overset{\alpha_{R}}{\to }Q+F+R$	$\frac{\alpha_{R}}{\Omega }X_{Q}X_{F}$
$R\overset{\beta_{R}}{\to }$	*β*_*R*_*X*_*R*_
$R+R\overset{k_{f_{2}}}{\to }R:R$	$\frac{k_{f_{2}}}{\Omega }X_{R}\left(X_{R}-1\right)$
$R:R\overset{\beta_{R:R}}{\to }R+R$	*β*_*R*: *R*_*X*_*R*: *R*_

Here $\alpha_{M}=\alpha_{P}=\beta_{M}=\beta_{P}=\frac{k_{f_{1}}}{\Omega }=k_{b_{1}}=\frac{k_{f_{2}}}{\Omega }=k_{b_{2}}=1\;m^{-1}$ and $\alpha_{E}\Omega =\beta_{E}=\frac{\alpha_{Q}}{\Omega }=\beta_{Q}=\alpha_{F}\Omega =\beta_{F}=\frac{\alpha_{R}}{\Omega }=\beta_{R}=\frac{1}{\epsilon }\;m^{-1}$. Ω represents the volume of the system and *ϵ* ≪ 1.

Among slow reactions, dimerization of *R* to *R*: *R* depends on the state of fast species *R*. Thus, we need to derive the QSS of the propensity function for the dimerization, $\frac{k_{f_{2}}}{\Omega }\langle X_{R}\left(X_{R}-1\right)|X_{P}\rangle$. This QSS can be derived using the FEEDME algorithm since all fast species, *E*, *Q*, *F* and *R* constitute a feedforward network (Fig 3):

$$\begin{array}{c} \frac{k_{f_{2}}}{\Omega }\langle X_{R}\left(X_{R}-1\right)|X_{P}\rangle =k_{f_{2}}X_{P}\left(\frac{X_{P}}{\Omega }+\frac{1}{\Omega }\frac{7X_{P}/\Omega +4}{8}+\frac{1}{\Omega^{2}}\frac{2X_{P}/\Omega +3}{9}\right) \end{array}$$ (6)

By substituting this QSS of the propensity function into the original propensity function for the dimerization, we can derive a reduced model (Fig 3 below), which is referred to as the EMB model for this example. On the other hand, the following approximate moment under the moment closure assumption (〈*X*_*i*_*X*_*j*_〉 = 〈*X*_*i*_〉〈*X*_*j*_〉) can be used:

$$\begin{array}{c} \frac{k_{f_{2}}}{\Omega }\langle X_{R}\left(X_{R}-1\right)|X_{P}\rangle \approx \frac{k_{f_{2}}}{\Omega }\langle X_{R}|X_{\;P}\rangle \langle X_{R}-1|X_{P}\rangle =k_{f_{2}}X_{P}\left(\frac{X_{P}-1}{\Omega }\right). \end{array}$$ (7)

The reduced model, where the approximate moment is substituted by $\frac{k_{f_{2}}}{\Omega }\langle X_{R}\left(X_{R}-1\right)|X_{P}\rangle$, is referred to as the AMB model for this example. Again, when Ω → ∞ and thus the system becomes closer to the deterministic system, the approximate moment Eq (7) and the exact moment Eq (6) become equivalent.

**Fig 4:**
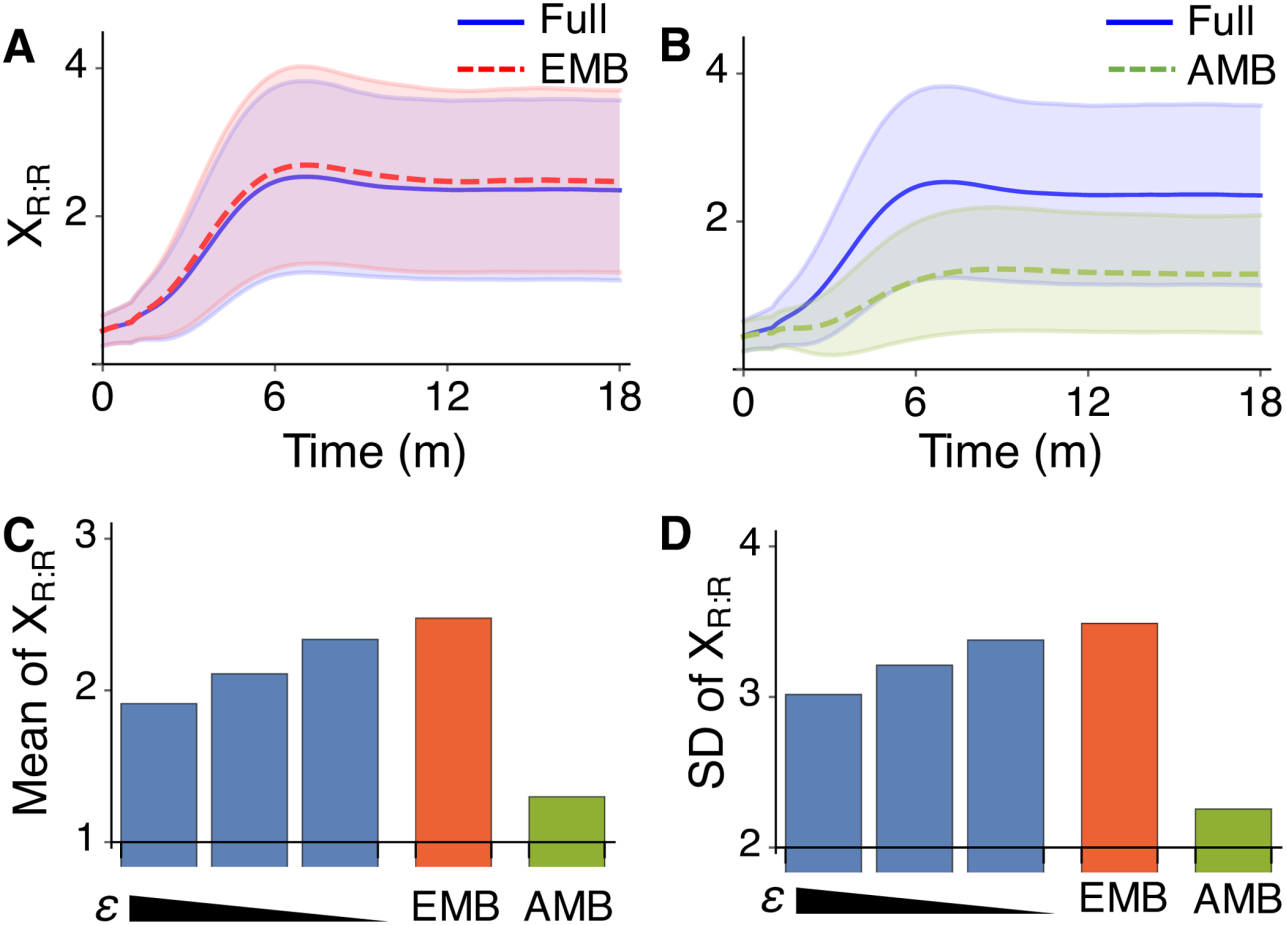
The EMB model provides more accurate approximation of the transcriptional negative feedback loop model than AMB model. (A-B) Trajectories of the full model with *ϵ* = 0.01 and the EMB model (A) and the AMB model (B). The lines and colored ranges represent *E*(*X*_*R*: *R*_) and *E*(*X*_*R*: *R*_) ± *SD*(*X*_*R*: *R*_)/2 of 10^4^ stochastic simulations. Here *X*_*i*_(0) = 0. (C-D) Mean (C) and standard deviation (D) of steady state distribution of the full model with varying *ϵ* = 0.1, 0.05, 0.01, the EMB model and the AMB model.

When Ω is instead small, so that the system has large fluctuations, the AMB model fails to approximate the full model with *ϵ* = 0.01 (Fig 4B). On the other hand, the EMB model provides an accurate approximation (Fig 4A). Specifically, the mean and standard deviation of trajectories of the full model are accurately captured by the EMB model, but not by the AMB model. Furthermore, as *ϵ* decreases so that the feedforward subnetwork becomes faster, the mean and standard deviation of the slow species *R*: *R* at the steady state of the full model converge to those of the EMB model, but not to those of the AMB model (Fig 4C and 4D). Our example example illustrates how FEEDME can be used to reduce a multiscale BRN whose fast subnetwork is feedforward. In fact, FEEDME can be used for the reduction of more general BRNs as long as their fast subnetwork has a linear moment closure property and thus the QSS can be calculated using Eq (15) recursively.

### Reduction of a stochastic system with a fast reversible binding

Next, we consider a different class of multiscale BRNs, those whose fast subnetwork is a complex balanced network. When a network follows mass action kinetics, the network is complex balanced if it is weakly reversible and its deficiency is zero. Weak reversibility means that if there is a path from a given component to another, there is also a reverse (possibly different) path back from the second component to the first one. The deficiency is an integer calculated as the number of components or nodes minus the number of connected components, minus the rank of stoichiometry matrix (see Methods for details). Complex balanced networks can include fast reversible bindings, which are not allowed in the definition of feedforward networks.

**Fig 5:**
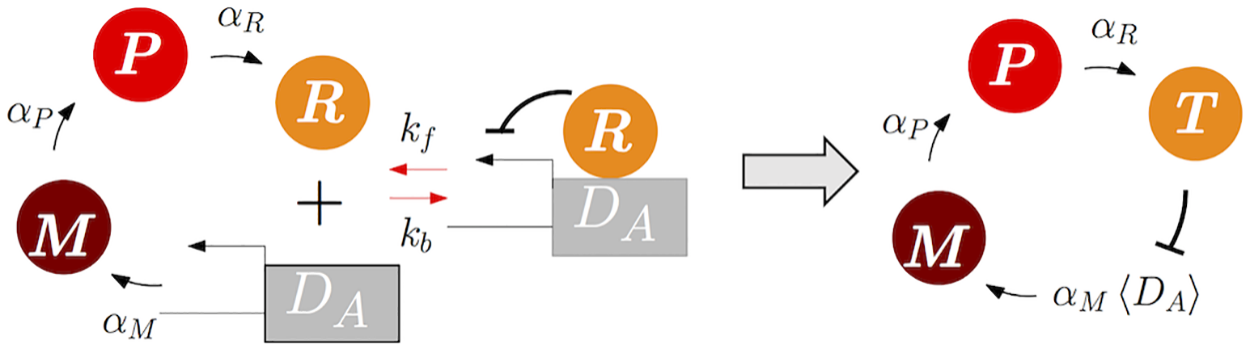
Model diagram of a genetic oscillator with a fast reversible binding. In the diagram of the full model (left), red arrows indicate the fast reversible binding and unbinding reactions. Note that the fast species *R*, *D*_*A*_ and their complex form a complex balanced network. In the diagram of the reduced model (right), which consists solely of slow species and reactions, 〈*D*_*A*_〉 represents the conditional moment $\langle X_{D_{A}}|X_{T}\rangle$, where $X_{T}\;=\;X_{R}\;+\;X_{D_{R}}$. In both of the diagrams, degradation reactions are not shown, for simplicity.

We next study multiscale BRNs whose fast subnetwork is complex balanced. This fast subnetwork can be accurately reduced using a formula for stationary moments given later, Eq (19), which is valid even in the presence of conservation laws (see Methods for details) [61, 62]. We illustrate this with an example of a transcriptional negative feedback loop (Fig 5 and Table 3), where the transcription of mRNA (*M*) occurs proportionally to active promoter (*D*_*A*_) and then *M* is translated into protein (*P*), which promotes the production of the repressor (*R*). The repressor reversibly binds with *D*_*A*_ to form repressed promoter complex (*D*_*R*_), thus completing a transcriptional negative feedback loop. Since this model can generate oscillations, it has been widely used to study circadian rhythms [66–70].

**Table 3:** Reactions and propensity functions in the genetic oscillator (Fig 5).

Reactions	Propensity functions
$D_{A}\overset{\alpha_{M}}{\to }D_{A}+M$	$\alpha_{M}X_{D_{A}}$
$M\overset{\beta_{M}}{\to }\phi$	*β*_*M*_*X*_*M*_
$M\overset{\alpha_{P}}{\to }M+P$	*α*_*P*_*X*_*M*_
$P\overset{\beta_{P}}{\to }\phi$	*β*_*P*_*X*_*P*_
$P\overset{\alpha_{R}}{\to }P+R$	*α*_*R*_*X*_*P*_
$R\overset{\beta_{R}}{\to }\phi$	*β*_*R*_*X*_*R*_
$D_{R}\overset{\beta_{R}}{\to }D_{A}$	$\beta_{R}X_{D_{R}}$
$D_{A}+R\overset{k_{f}}{\to }D_{R}$	$\frac{k_{f}}{\Omega }X_{D_{A}}X_{R}$
$D_{R}\overset{k_{b}}{\to }D_{A}+R$	$k_{b}X_{D_{R}}$

Here *α*_*M*_ = 10 *h*^−1^ and *α*_*P*_ = *α*_*R*_ = *β*_*M*_ = *β*_*P*_ = *β*_*R*_ = 1 *h*^−1^. $\frac{k_{f}}{\Omega }=\frac{K}{\Omega }\frac{1}{\epsilon }\;h^{-1}$ and $k_{b}=\frac{1}{\epsilon }\;h^{-1}$. $\frac{K}{\Omega }=\frac{10^{3}}{625}$ is an inverse of a dissociation constant whose unit is the number of molecules. Total number of promoter ($X_{D_{{}_{T}}}:=X_{D_{A}}+X_{D_{R}}$) is 10. Ω represents the volume of the system and *ϵ* ≪ 1.

When binding and unbinding reactions are fast, *D*_*A*_, *D*_*R*_ and *R* become fast species. Among slow reactions, the transcription depends on the states of fast species *D*_*A*_, and thus we need to derive the QSS of the propensity function, $\alpha_{M}X_{D_{A}}$ for the reduction. The exact QSS can be derived since the subnetwork of fast species with a following fast reversible binding is a complex balanced network as described in Eq (S1) (see S1 Appendix for details):

$$\begin{array}{c} D_{A}+R\;\overset{k_{f}}{\underset{k_{b}}{⇌}}D_{R}. \end{array}$$ (8)

Note that there is a conservation law as the total number of promoter ($X_{D_{{}_{T}}}:=X_{D_{A}}+X_{D_{R}}$) is conserved. Furthermore, the total repressor species ($X_{T}:=X_{R}+X_{D_{R}}$) is a slow species as it is not affected by the fast binding and unbinding reactions (i.e. the fast binding and unbinding reactions in *R* and *D*_*R*_ are canceled). As *T* evolves slowly, although *T* is not constant on a slow timescale, it can be treated as constant or conserved on a fast timescale. Thus, on a fast timescale when slow species are treated as constant, the complex balanced network Eq (8) obeys two conservation laws: *D*_*T*_ = *D*_*A*_ + *D*_*R*_ and *T* = *R* + *D*_*R*_. For such a complex balanced network with conservations, we can derive the stationary conditional moment of any species using Eq (19). In particular, when $p=X_{D_{T}}=10$ and *n* = *X*_*T*_, our code based on Eq (19) and Eq (S3) derives $\langle X_{D_{A}}|X_{T}\rangle$ as follows (see S1 Appendix for details).

$$\begin{array}{lll} \langle X_{D_{A}}|X_{T}\rangle & = & f(X_{T})/g(X_{T}).\\ f(X_{T}) & = & 10+2.58274\cdot 10^{16}X_{T}-7.04208\cdot 10^{16}X_{T}{}^{2}+7.62519\cdot 10^{16}X_{T}{}^{3}\\ & & -4.36905\cdot 10^{16}X_{T}{}^{4}+1.46859\cdot 10^{16}X_{T}{}^{5}-2.99466\cdot 10^{15}X_{T}{}^{6}\\ & & +3.64482\cdot 10^{14}X_{T}{}^{7}-2.43525\cdot 10^{13}X_{T}{}^{8}+6.87195\cdot 10^{11}X_{T}{}^{9},\\ g(X_{T}) & = & 1-3.72234\cdot 10^{17}X_{T}+1.0559\cdot 10^{18}X_{T}{}^{2}-1.21071\cdot 10^{18}X_{T}{}^{3}\\ & & +7.50742\cdot 10^{17}X_{T}{}^{4}-2.81072\cdot 10^{17}X_{T}{}^{5}+6.65138\cdot 10^{16}X_{T}{}^{6}\\ & & -1.00206\cdot 10^{16}X_{T}{}^{7}+9.32029\cdot 10^{14}X_{T}{}^{8}-4.87908\cdot 10^{13}X_{T}{}^{9}\\ & & +1.09951\cdot 10^{12}X_{T}{}^{10} \end{array}$$

By substituting this QSS of the propensity function into $\alpha_{M}X_{D_{A}}$, we can derive a reduced model (Fig 5), which is referred to as the EMB model for this example. On the other hand, previous studies used the deterministic QSS as an approximation [48, 49]:

$$\langle X_{D_{A}}|X_{T}\rangle \begin{array}{c} \approx \frac{\left(X_{D_{T}}-X_{T}-\Omega /K+\sqrt{{\left(X_{D_{T}}-X_{T}-\Omega /K\right)}^{2}+4\Omega /K}\right)}{2} \end{array}.$$

**Fig 6:**
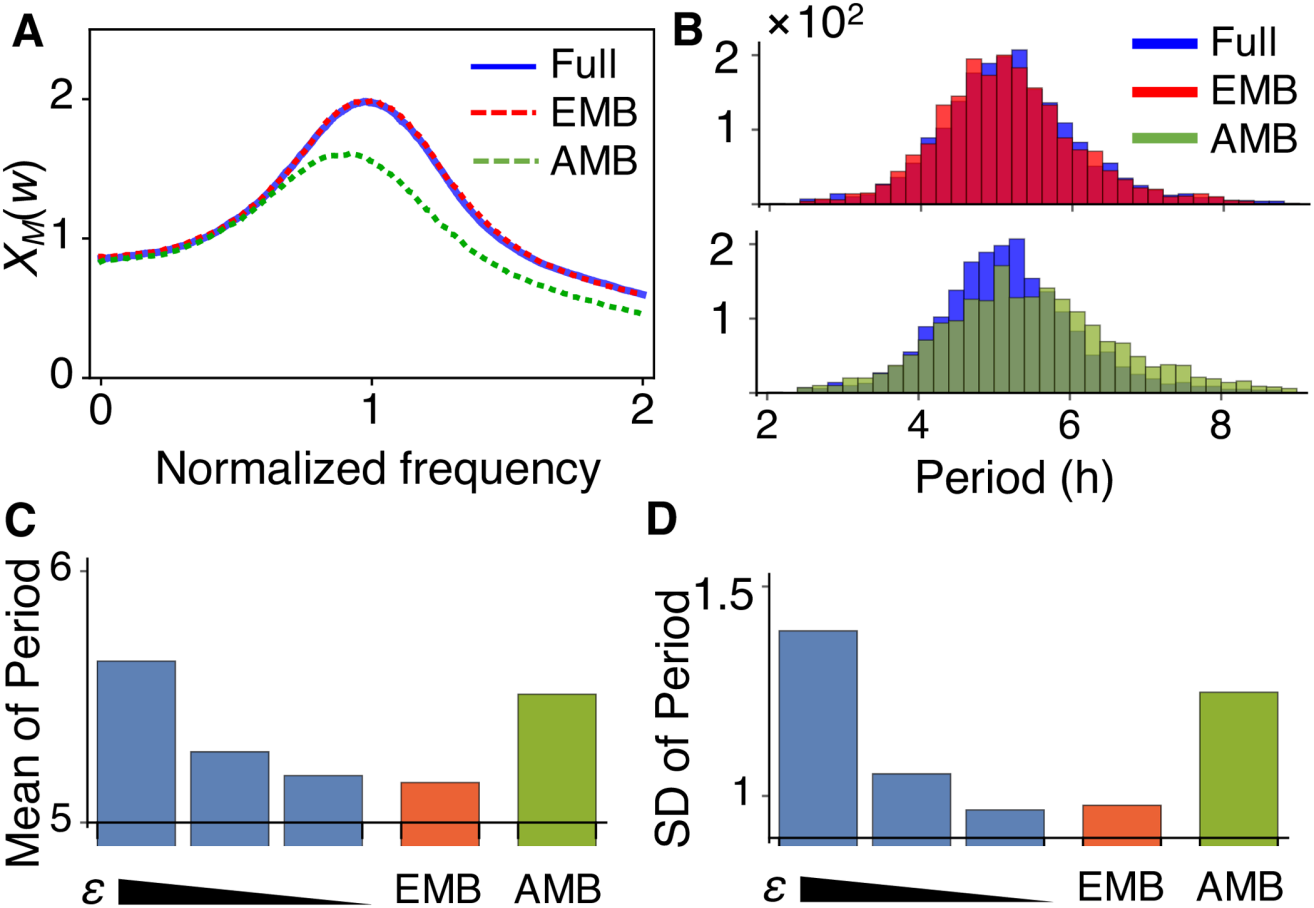
Period distributions of full and reduced models. (A) Fourier transforms of stochastic trajectories with about 10^4^ cycles of the EMB model, the AMB model and the full model with *ϵ* = 0.1. (B) After partitioning the trajectory of 10^4^ cycles into 2⋅10^3^ trajectories so that each trajectory consists of about 5 cycles, the autocorrelation of each trajectory is calculated to estimate the period of each trajectory. The period distribution of 2⋅10^3^ trajectories of the EMB model (above) better captures that of the full model with *ϵ* = 0.1 than that of the AMB model (bottom). (C-D) The mean and the standard deviation of period distributions of the EMB model, the AMB model, and the full model with *ϵ* = 10, 1, 0.1.

By substituting this approximating moment for $X_{D_{A}}$, we can derive another reduced model, which is referred to as the AMB model for this example. The deterministic QSS used in the AMB model is derived with the total QSSA, which uses the new slow variable (*T*) to improve the accuracy of the reduction. The stochastic reduction based on the total QSSA has been known to provide more accurate approximation for the stochastic simulations of the full model than the model based on the standard QSSA (see [22, 23, 48, 49] for details). However, the EMB model provides an even more accurate approximation (Fig 6A and 6B). Specifically, both Fourier transforms and the distribution of periods of simulated trajectories with the full model is more accurately captured by the EMB model than the AMB model. The mean and standard deviation of the periods of the full model approach to those of the EMB model, but not the AMB model as the reversible binding becomes faster (i.e. *ϵ* decreases) (Fig 6C and 6D). In summary, when fast species form a complex balanced network with conservation laws, we can find the stationary conditional moments of fast species using Eq (19) and thus derive a more accurate reduction of the stochastic system.

### Reduction of a stochastic system with a coupled fast competitive bindings

**Fig 7:**
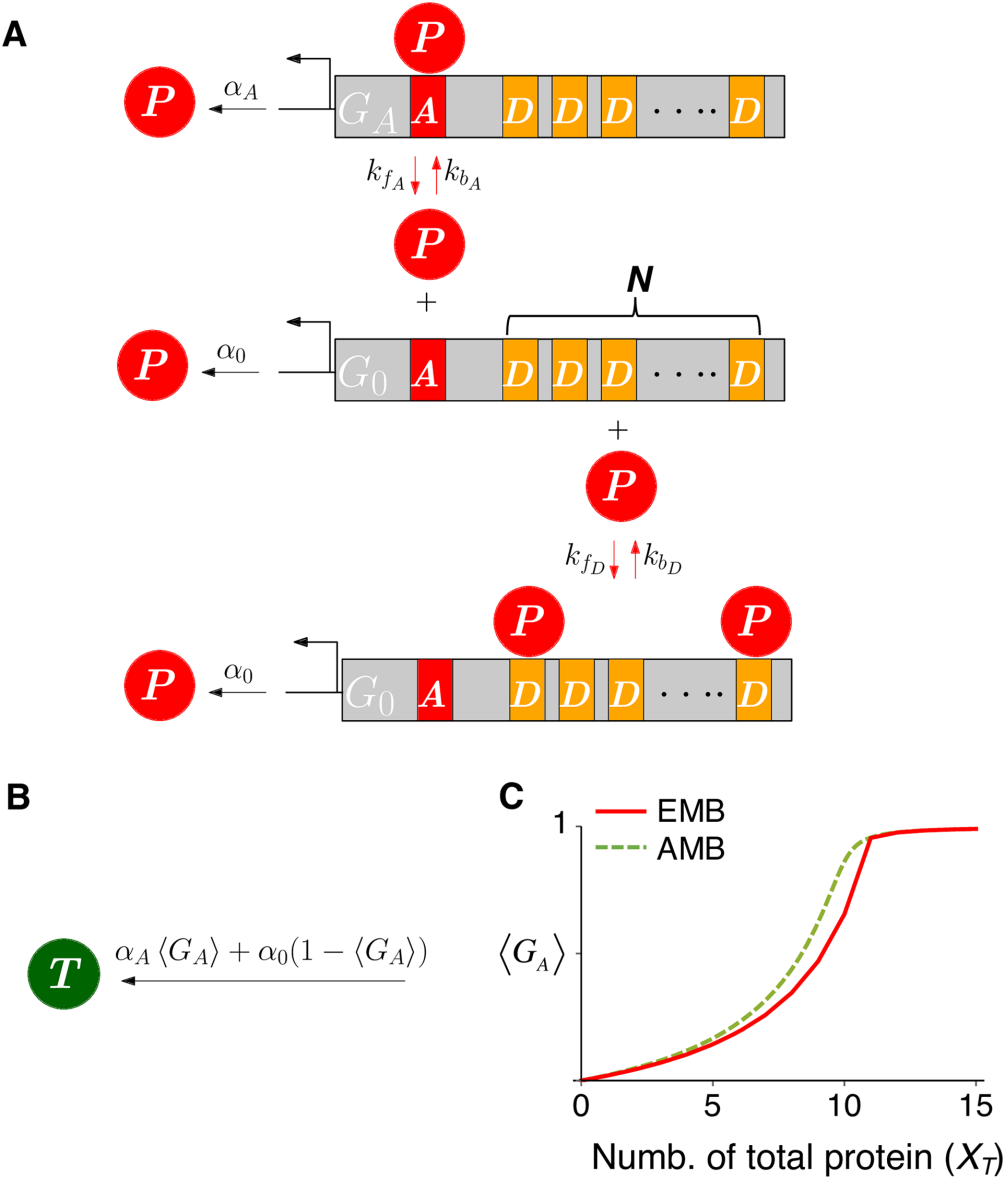
Model diagram of a positive feedback loop with decoys. (A) In the diagram of the full model, red arrows indicate the fast reversible binding and unbinding reactions between transcriptional factor (*P*) and either a regulatory promoter site (*A*) or one of *N* identical nonregulatory decoy binding sites (*D*). These two fast reversible bindings form a complex balanced network with species *P*, *G*_0_, *G*_*A*_, *D*, and *P*: *D* with conservations, $X_{G_{0}}+X_{G_{1}}=1$ and *X*_*D*_ + *X*_*P*: *D*_ = *N*. (B) The reduced model consists solely of a slow species, which is the total number of transcription factors *T* ($X_{T}\;=\;X_{P}+X_{G_{A}}+X_{P:D}$). 〈*G*_*A*_〉 represents stationary conditional moment, $\langle X_{G_{A}}|X_{T}\rangle$. In both of the diagrams (A and B), degradation reactions are not shown, for simplicity. (C) The exact moment $\langle X_{G_{A}}|X_{T}\rangle$, which is used for the EMB model and its approximation, which is used for the AMB model. When *X*_*T*_ < *N* = 10, they show a discrepancy.

Here we consider another multiscale stochastic system, a transcriptional positive feedback loop with decoys (Fig 7A and Table 4) whose fast timescale subnetwork consists of coupled competitive reversible bindings and is complex balanced. This was developed to investigate the influence of decoys on gene expression noise [71]. In the model, transcription factors (*P*) can bind to both a promoter site (*G*_0_) and *N* identical nonregulatory decoy binding sites (*D*). When *P* is bound to the promoter site, the gene becomes active (*G*_*A*_) and thus the transcriptional rate increases. While decoy is assumed to protect the transcription factor from degradation in the original model [71], here we assume that the transcriptional factor degrades equally regardless of its binding status (Table 4).

**Table 4:** Reactions and propensity functions in the positive feedback loop with decoys (Fig 7A).

Reactions	Propensity functions
$G_{0}\overset{\alpha_{0}}{\to }G_{0}+P$	$\alpha_{0}X_{G_{0}}$
$G_{A}\overset{\alpha_{A}}{\to }G_{A}+P$	$\alpha_{A}X_{G_{A}}$
$G_{0}+P\overset{k_{f_{A}}}{\to }G_{A}$	$\frac{k_{f_{A}}}{\Omega }X_{G_{0}}X_{P}$
$G_{A}\overset{k_{b_{A}}}{\to }G_{0}+P$	$k_{b_{A}}X_{G_{A}}$
$D+P\overset{k_{f_{D}}}{\to }D:P$	$\frac{k_{f_{D}}}{\Omega }X_{D}X_{P}$
$D:P\overset{k_{b_{D}}}{\to }D+P$	$k_{b_{A}}X_{D:P}$
$P\overset{\beta_{P}}{\to }\phi$	*β*_*P*_*X*_*P*_
$D:P\overset{\beta_{P}}{\to }D$	*β*_*P*_*X*_*D*: *P*_
$G_{A}\overset{\beta_{P}}{\to }G_{0}$	$\beta_{P}X_{G_{A}}$

**Fig 8:**
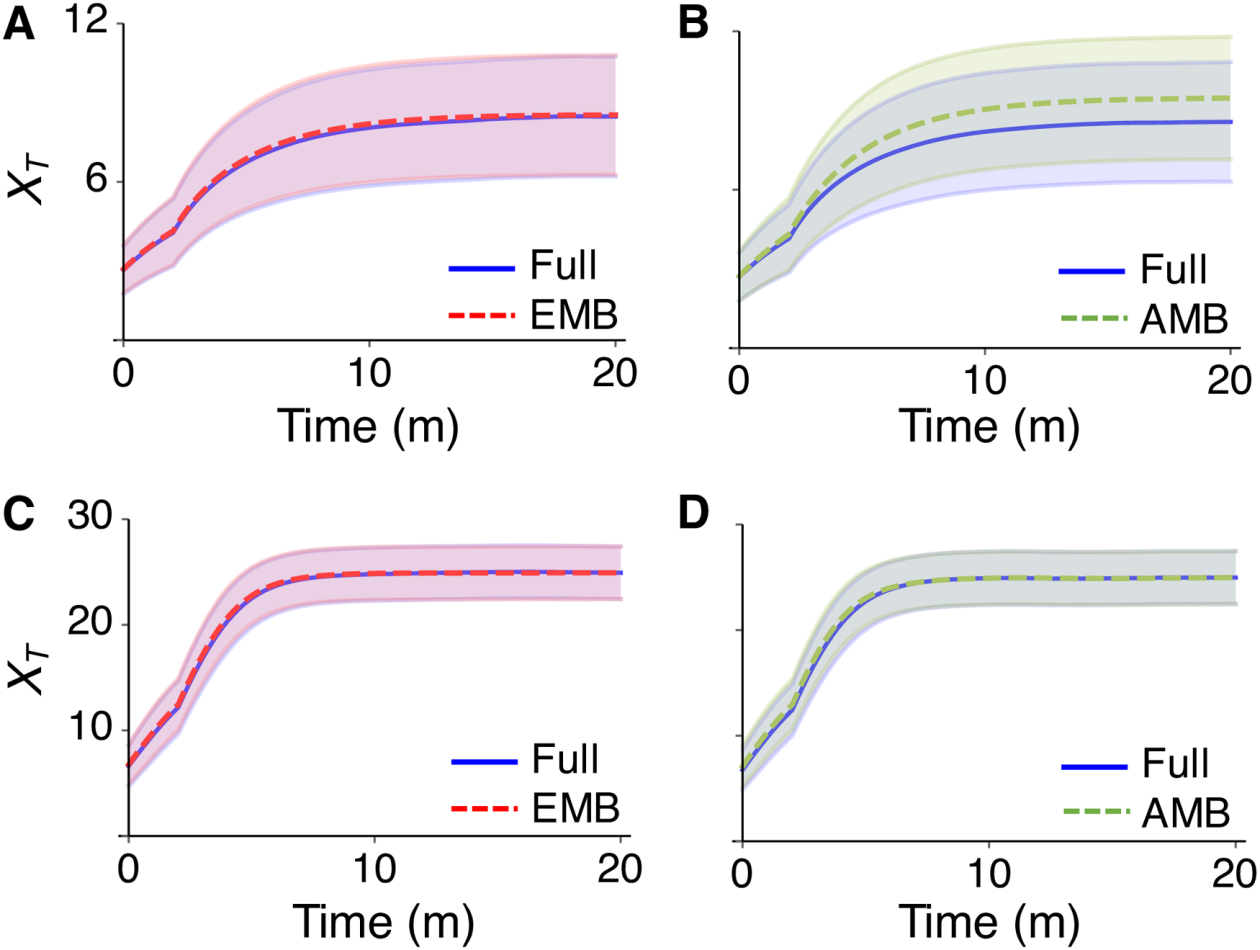
Trajectories of the full model, the EMB model and the AMB model of the positive feedback loop with decoys. (A-B) The lines and colored ranges represent *E*(*X*_*T*_) and *E*(*X*_*T*_) ± *SD*(*X*_*T*_)/2 of 10^4^ stochastic simulations when *α*_*A*_ = 10 and *α*_0_ = 4. Here *X*_*i*_(0) = 0. (C-D) When *α*_*A*_ = 20 and *α*_0_ = 8, so that overall *X*_*T*_ is greater than the total number of decoy sites (i.e *N* = 10), the AMB model also becomes accurate.

Here *α*_0_ = 4 *m*^−1^, *α*_*A*_ = 10 *m*^−1^ (Fig 8A and 8B) or *α*_0_ = 8 *m*^−1^, *α*_*A*_ = 20 *m*^−1^ (Fig 8C and 8D). $\frac{k_{f_{A}}}{\Omega }=2000\;m^{-1}$, $k_{b_{A}}=100\;m^{-1}$, $\frac{k_{f_{D}}}{\Omega }=10000\;m^{-1}$, $k_{b_{D}}=100\;m^{-1}$, *β*_*P*_ = 0.8 *m*^−1^. Total number of promoter ($X_{G_{0}}+X_{G_{A}}$) is 1. Total number of decoy sites (*N*: = *X*_*D*_ + *X*_*D*: *P*_) is 10. Ω represents the volume of the system.

Because reversible bindings between *P* and *G*_0_ or *D* are faster than other reactions, the following competitive bindings form the fast timescale subnetwork.

$$G_{0}+P\overset{k_{f_{A}}}{\underset{k_{b_{A}}}{⇌}}G_{A}$$

$$D+P\overset{k_{f_{D}}}{\underset{k_{b_{D}}}{⇌}}D:P$$

This subnetwork is a complex balanced network as described in Eq (S4) in S1 Appendix. This network obeys two conservation laws: total gene number ($X_{G_{0}}+X_{G_{A}}$) and total decoy sites (*X*_*D*_ + *X*_*D*: *P*_) are 1 and 10, respectively. Furthermore, total transcriptional factors (*T*: = *P* + *G*_*A*_ + *D*: *P*) slowly evolve as they are not affected by fast binding and unbinding reactions. Thus, on a fast timescale, *T* can be treated as a constant or conserved. Since this is a complex balanced network, we can use Eq (19) to derive any of the stationary moments, subject to the three conservations. In particular, Eq (S6) in S1 Appendix with *n*_*A*_ = *X*_*T*_ and *n*_*C*_ = *X*_*D*_ + *X*_*D*: *P*_ = 10 yields $\langle X_{G_{A}}|X_{T}\rangle$, which can be calculated with the code provided in this work (Fig 7C). This derives the exact QSS of slow propensity functions, $\alpha_{P}X_{G_{A}}$ and $\alpha_{0}X_{G_{0}}=\alpha_{0}\left(1-X_{G_{A}}\right)$, describing the production of the slow species *T*: $\alpha_{A}\langle X_{G_{A}}|X_{T}\rangle$ and $\alpha_{0}\left(1-\langle X_{G_{A}}|X_{T}\rangle \right)$. By using the exact QSS, the reduced model, which is solely determined by the slow species *T* can be derived (Fig 7B). This reduced model is referred to as the EMB model for this example. On the other hand, the previous study [71] used the following approximate moment:

$$\langle X_{G_{A}}|X_{T}\rangle \approx \frac{\left(X_{T}-K_{D}/\Omega -N+\sqrt{{\left(X_{T}-K_{D}/\Omega -N\right)}^{2}+4K_{D}X_{T}/\Omega }\right)}{2K_{A}/\Omega +(X_{T}-K_{D}/\Omega -N+\sqrt{{\left(X_{T}-K_{D}/\Omega -N\right)}^{2}+4K_{D}X_{T}/\Omega })},$$ (9)

where $K_{D}=k_{b_{D}}/k_{f_{D}}$ and $K_{A}=k_{b_{A}}/k_{f_{A}}$. The reduced model based on this approximate moment is referred to as the AMB model for this example. The previous study found that the accuracy of the AMB model decreases as the decoy binding becomes tighter (i.e. smaller *K*_*D*_) [71]. Thus, we investigated whether the EMB model can provide an accurate approximation even when the *K*_*D*_ is small. Despite the small value of *K*_*D*_, the EMB model provides an accurate approximation in contrast to the AMB model (Fig 8A and 8B). This describes that the inaccuracy of the AMB model when *K*_*D*_ is small stems from the inaccuracy of the approximate moment Eq (9). Indeed, when we compared the exact moment and the approximate moment (Fig 7C), we characterized the discrepancy between them. In particular, the discrepancy mainly occurs when *T* is less than the total number of decoy sites (i.e. 10). Thus, we hypothesized that the accuracy of the AMB model increases as *T* increases. To investigate this, we increased the transcription rates *α*_*A*_ and *α*_0_ so that overall level of *T* increases. In this case, the accuracy of the AMB model considerably increases (Fig 8C and 8D). This indicates that by comparing the exact moment and the approximate moment, we can find validity conditions for the AMB model and thus when the stochastic analysis based on the approximation Eq (9) in the previous study [71] is valid (e.g. when *T* ≫ *N*).

## Discussion

Stochastic multiscale BRNs can be reduced by replacing the fast species with their QSS, which accelerates stochastic simulations. For the reduction, approximate QSS of fast species are frequently used in the presence of nonlinear reactions [8, 14–27]. However, the validity conditions for such approximations have not been fully understood or are often difficult to test [45–50]. In this work, we have demonstrated that a fairly large class of nonlinear BRNs can be reduced by deriving the exact QSS of fast species rather than the approximate QSS even in the presence of nonlinear reactions. Specifically, when fast species constitute either a feedforward network or a complex balanced network with conservations, their exact QSS can be derived using our computational packages based on the Eq (15), Eq (S3), or Eq (S6) (S1 Appendix) [60, 62]. Reduced models in this way accurately approximate the stochastic dynamics of the original full models as long as disparate timescales exist (Figs 2, 4, 6 and 8).

As complex balanced networks with conservations include various types of reversible bindings, they are frequently observed as a fast subnetwork of gene regulatory networks where fast bindings occur between molecules such as transcriptional factors and RNA polymerase and regulatory or non-regulatory sites of genes [57]. Complex balanced networks are also embedded in many signalling networks such as receptor-ligand signal pathways in response to various stimuli such as heat [26, 72], inflammation [73], and blood glucose [74]. Furthermore, a feedforward network is one of fundamental motifs of gene and protein regulatory networks [64, 65]. Interestingly, a feedforward network is also observed in the networks of neuronal cells where stochastic multiscale dynamics of ion channels exist [75–78]. Extension of our work to such multiscale stochastic neuronal networks would be an interesting direction for future work. Another interesting future work would be to investigate other classes of nonlinear BRNs whose exact moments can be derived. In particular, our computational package, FEEDME can be used to derive the exact moment for any BRNs which have linear moment closure property as well as the feedforward network.

Various approximation schemes for the QSS of fast species have been usually tested by comparing the simulated distributions or trajectories of a reduced model and an original full model [8, 14–27]. Such a test is computationally expensive because it requires stochastic simulations of a multiscale full model for many times. Such high computational cost is prohibitive when testing the accuracy of the approximation for complex examples or a wide range of parameters. On the other hand, if an exact QSS is known, the accuracy of the approximation can be tested easily by comparing the exact QSS and the approximate QSS as seen in Fig 7C. Furthermore, in this way, we can understand more intuitively under which conditions the approximation becomes accurate or not (Fig 8). Thus, non-linear feedforward networks or complex balanced networks can be an effective test platform for various approximate schemes such as normal or log-normal moment closure [52].

## Methods

### Chemical master equation

A BRN consists of a finite set of reactions $R=\{R_{j},j=1,2,\ldots ,m\}$ acting on species, $S=\{S_{i},\;i=1,2,\ldots n\}$. By defining

$$\begin{array}{c} S_{i}\left(t\right)=\;\text{the}\;\text{number}\;\text{of}\;\text{species}\;S_{i}\;\text{at}\;\text{time}\;t\; \end{array}$$

we can denote the probability that the state of the system, *S* = (*S*_1_, …, *S*_*n*_)′ equals $k=\left(k_{1},\ldots ,k_{n}\right)\in Z_{\geq 0}^{n}$ at *t* as

$$\begin{array}{c} p_{k}\left(t\right)=P\;\left[S(t)=k\right]. \end{array}$$

For reactions,

$$\begin{array}{c} R_{j}:\;\sum_{i=1}^{n}a_{ij}S_{i}\;⟶\;\sum_{i=1}^{n}b_{ij}S_{i}\;,\;j\in \left\{1,2,\ldots ,m\right\}, \end{array}$$ (10)

where *a*_*ij*_ and *b*_*ij*_ are *stoichiometry coefficients*, their stochastic behaviors are described by propensity functions:

$$\begin{array}{c} \rho_{j}:Z_{\geq 0}^{n}\to R_{\geq 0},\;j=1,\ldots ,m,\;\text{with}\;\rho_{j}\left(0\right)=0 \end{array}$$

Specifically, *ρ*_*j*_(*k*_1_, …, *k*_*n*_)*dt* is the probability that reaction *R*_*j*_ takes place, in a short interval of length *dt*, provided that the complete state was *S* = (*k*_1_, …, *k*_*n*_) at the beginning of the interval. Mass action kinetics based on collision theory assumes that the propensity function is proportional to the number of ways in which species can combine to form the *j*th source complex (see [79]) when temperature and volume are constant, and the system is well-mixed:

$$\rho_{j}(k)=\kappa_{j}\prod_{i=1}^{n}\left({}_{a_{ij}}^{k_{i}}\right)\;\;\;\;\;j=1,\ldots ,m.$$ (11)

where $\left({}_{a_{ij}}^{k_{i}}\right)$ is the combinatorial number and is zero by definition when any *k*_*i*_ < *a*_*ij*_. The coefficients *κ*_*j*_ are non-negative “kinetic constants” represents quantities related to the volume, shapes of the reactants, chemical and physical information, and temperature. In particular, $\kappa_{j}=\frac{\kappa_{j}^{\prime }}{\Omega^{\sum_{i=1}^{n}a_{ij}-1}}$ when $\kappa_{j}^{\prime }$ is macroscopic reaction rate in terms of concentration and Ω is the volume of system.

Using the propensity functions, we can derive a *Chemical Master Equation (CME)*, which is the differential form of the Chapman-Kolmogorov forward equation for *p*_*k*_(*t*):

$$\begin{array}{c} \frac{dp_{k}}{dt}\;=\;\sum_{j=1}^{m}\rho_{j}\left(k-\gamma_{j}\right)\;p_{k-\gamma_{j}}-\sum_{j=1}^{m}\rho_{j}\left(k\right)\;p_{k}\;,\;k\in Z_{\geq 0}^{n} \end{array}$$ (12)

where *γ*_*j*_ is a vector whose *i*th component is *b*_*ji*_−*a*_*ji*_, which represents the net change in the number of *S*_*i*_ each time that *R*_*j*_ occurs. Note that the propensity function *ρ*_*j*_ has the property that *ρ*_*j*_(*k*−*γ*_*j*_) = 0 unless *k* ≥ *γ*_*j*_ (coordinatewise inequality). There is one equation for each $k\in Z_{\geq 0}^{n}$, so this is an infinite system of linked equations. When discussing the CME, we will assume that an initial probability vector *p*(0) has been specified, and that there is a unique solution of Eq (12) defined for all *t* ≥ 0. We do not discuss existence and uniqueness results, which are subtle. See [80] for details.

### Feedforward networks

#### Derivatives of moments are expressed as linear combinations of moments

Here, we review how to derive equations for derivatives of moments with arbitrary orders using simple algebra of polynomials. First, note that each rate *ρ*_*j*_(*k*) as in Eq (11) can be expanded into a polynomial in which each variable *k*_*i*_ has an exponent less or equal to *a*_*ij*_ for suitably redefined coefficients $\kappa_{c_{j}}$’s:

$$\begin{array}{c} \rho_{j}\left(k\right)=\sum_{c_{j}\leq a_{j}}\kappa_{c_{j}}\;k^{c_{j}}, \end{array}$$ (13)

where $k^{c_{j}}=k_{1}^{c_{1j}}\ldots k_{n}^{c_{nj}}$ and “≤” is understood coordinatewise. For a monomial function $M:Z_{\geq 0}^{n}\to R$:

$$\begin{array}{c} M\left(k\right)=k^{u}=k_{1}^{u_{1}}k_{2}^{u_{2}}\ldots k_{n}^{u_{n}}, \end{array}$$

the expectation of the random variable *M*(*S*) is

$$\begin{array}{c} E\left[M(S(t))\right]=\sum_{k\in Z_{\geq 0}^{n}}p_{k}\left(t\right)\;M\left(k\right)\;, \end{array}$$

since $p_{k}\left(t\right)=P\;[S(t)=k]$. After taking the derivative of this equation and substituting CME Eq (12) to $\frac{dp_{k}}{dt}$, the following can be derived (see [81] for more details):

$$\begin{array}{c} \frac{d}{dt}E\left[M(S(t))\right]\;=\;\sum_{j=1}^{m}E\left[\rho_{j}\left(S\left(t\right)\right)\;\Delta_{\gamma_{j},M}\left(S\left(t\right)\right)\right], \end{array}$$ (14)

where Δ_*γ*_*j*_, *M*_(*k*): = *M*(*k* + *γ*_*j*_) − *M*(*k*). For instance,

$$\begin{array}{c} \Delta_{\left(1,0\right),k_{1}^{2}k_{2}}\left(k_{1},k_{2}\right)={\left(k_{1}+1\right)}^{2}k_{2}-k_{1}^{2}k_{2}=2k_{1}k_{2}+k_{2}. \end{array}$$

Note that $k_{1}^{2}k_{2}$ is canceled. Such cancelation in *M*(*k* + *γ*_*j*_)−*M*(*k*) leads to

$$\begin{array}{c} \Delta_{\gamma_{j},M}\left(k\right)=\sum_{\nu \in I(u,j)}\;d_{\nu }k^{\nu } \end{array}$$

for appropriate coefficients *d*_*ν*_, where

$$I\left(u,j\right):=\{\nu \in {\mathbb{Z}}_{\geq 0}^{n}|\begin{array}{c} \nu =u-\mu ,\;u\geq \mu \neq 0\\ \mu_{i}=0\text{ for each }i\text{ such that }\gamma_{ij}=0 \end{array}\}.$$

Combining this with Eq (13), the derivative of the moment of order *u* = (*u*_1_, …, *u*_*n*_) can be described as a linear combination of other moments (*ν* + *c*_*j*_):

$$\begin{array}{c} \frac{d}{dt}E\left[S{\left(t\right)}^{u}\right]=\sum_{j=1}^{m}\;\sum_{c_{j}\leq a_{j}}\;\sum_{\nu \in I(u,j)}d_{\nu }\kappa_{c_{j}}\;E\left[S{\left(t\right)}^{\nu +c_{j}}\right]. \end{array}$$ (15)

To solve this equation, we need to know all $E[S{\left(t\right)}^{\nu +c_{j}}]$, which typically results in an infinite set of coupled linear ordinary differential equations of moments. Often, for given a particular moment of order *u* of interest, there is a finite set of moments, including the desired one, that satisfies a finite set of differential equations (i.e. *linear moment closure*). Specifically, a given moment of order *u* is under linear moment closure when there exist *x*(*t*) = {*u*_1_, …, *u*_*N*_}, where *u*_1_ = *u*, and $A\in R^{N\times N}$ such that $\dot{x}\left(t\right)=Ax\left(t\right)$ for all *t* ≥ 0. The matrix *A* can be identified using Eq (15) recursively. It is well known that if all reactions have order 0 or 1, any moments are under a linear moment closure. Recent work found that such linear moment closure can be obtained for more general class of models such as nonlinear feedforward networks or some non-feedforward networks with conservations laws [60].

#### Linear moment closure property of feedforward network

A BRN is a *feedforward network* if its *n* species can be partitioned into *p* layers, in such a way that each reaction whose order is higher than one and involves species in layer *π* must have the form:

$$\begin{array}{c} a_{i_{1}j}S_{i_{1}}+\ldots a_{i_{q}j}S_{i_{q}}\;⟶\;a_{i_{1}j}S_{i_{1}}+\ldots a_{i_{q}j}S_{i_{q}}+b_{i_{q+1}j}S_{i_{q+1}}+\ldots b_{i_{q+q^{\prime }}j}S_{i_{q+q^{\prime }}} \end{array}$$

where all the species $S_{i_{1}},\ldots ,S_{i_{q}}$ belong to layers having indices <*π*, and the species $S_{i_{q+1}},\ldots ,S_{i_{q+q^{\prime }}}$ are in layer *π*. In other words, multimers of species in “previous” layers can “catalyze” the production of species in the given layer, but are not affected by these reactions. This can be summarized by saying that for reactions at any given layer *π*, the only species that appear as reactants in nonlinear reactions are those in layers <*π* and the only ones that can change are those in layer *π*.

It was shown in [60] that given any desired moment *u* of the feedforward network, there is a finite linear differential equation $\dot{x}\left(t\right)=Ax\left(t\right)$ for a suitable set of *N* moments

$$\begin{array}{c} x\left(t\right):={\left(E\left[S^{u_{1}}\left(t\right)\right],\ldots ,E\left[S^{u_{N}}\left(t\right)\right]\right)}^{\prime }, \end{array}$$

which contains the moment *u* of interest. In practice, we simply compute Eq (15) starting from a desired moment, then recursively apply the same rule to the moments newly appearing in the right-hand side, and so forth until no new moments appear. The integer *N* at which the system closes might be very large, but the procedure is guaranteed to stop. An estimation of the integer *N* can be done based on the solution of a linear program whose structure depends on the network. This is discussed in detail in [60]. To help performing this recursive calculation, we provide a computational package, FEEDME (FEEDforward Moment Equations) in this work. Then, the stationary moments can be computed by simply solving *Ax* = 0, which allows for the reduction of multi-scale feedforward network. For instance, the example in Fig 3 requires, for the computation of the conditional second moment of *R*, the following moments (*N* = 24): 0002, 0001, 0110, 0111, 0010, 0011, 0220, 1100, 1101, 0020, 0100, 0101, 0120, 0210, 1000, 1001, 1200, 1210, 0200, 1010, 1110, 2200, 2000, 2100, where we are using the convention that the string “*ijkl*” represents the expectation of *E*^*i*^ *F*^*j*^ *Q*^*k*^ *R*^*l*^. (The sample FEEDME.m script produces this output). The highest-order moments have order 4 (0220 and 2200). We also remark that non-feedforward networks also lead to the linear moment closure, provided that conservation laws ensure that variables appearing on nonlinear reactions take only a finite set of possible values (see [60]). Note that FEEDME can be used to derive moment equations for any BRNs who have linear moment closure property as well as the feedforward network.

### Complex balanced networks

#### Stationary moments of complex balanced networks with conservations

The large volume or thermodynamic limit of the CME is a deterministic system [82, 83]. For this associated deterministic network, a steady state $\bar{\lambda }\in R_{>0}^{n}$ is complex-balanced if, *for each individual* complex c∈C, the rate at which *c* is produced is equal to the rate at which it is consumed; i.e., outflows of *c* and inflows of *c* balance out.

Foundational results in deterministic complex balanced network theory were obtained by Horn, Jackson, and Feinberg (see [84, 85]). One of the key theorems is that a complex balanced steady state exists if the network is *weakly reversible* and have *deficiency zero*. Weak reversibility means that each connected component of the reaction graph must be strongly connected (i.e. if there is a direct path from a component to another, the vice versa is true). The deficiency is computed as *n*_*c*_ − *ℓ* − *r*, where *n*_*c*_ is the number of complexes, *r* is the rank of the matrix Γ, and *ℓ* is the number of “linkage classes” (connected components of the reaction graph). One of the most interesting features of this theorem is that no assumption needs to be made about the kinetic constants (Of course, the steady state will depend on kinetic constants). We refer the reader to the citations for details on deficiency theory, as well as, of interest in the present context, several examples discussed in [62]. The theorems for weakly reversible deficiency zero networks are actually far stronger, and they show that *every* possible steady state of the corresponding deterministic network is complex balanced, and also that they are asymptotically stable relative to stoichiometry classes.

The connection between the deterministic complex balanced steady state and the solutions of steady state CME (ssCME) was a beautiful observation made in [61], but can be traced to the “nonlinear traffic equations” from queuing theory, described in Kelly’s textbook [86], Chapter 8 (see also [87] for a discussion). Specifically, when the steady state vector, $\bar{\lambda }\in R_{>0}^{n}$ is complex-balanced,

$$\begin{array}{c} \Pi \;=\;\left\{p_{k}=\frac{{\bar{\lambda }}^{k}}{k!}\;,\;\;k\in Z_{\geq 0}^{n}\right\} \end{array}$$ (16)

becomes a solution of the ssCME equations. The elements of Π add up to:

$$\begin{array}{c} \sum_{k\in Z_{\geq 0}^{n}}p_{k}\;=\;\sum_{k_{1}=0}^{\infty }\ldots \sum_{k_{n}=0}^{\infty }\;\frac{{\bar{\lambda }}_{1}^{k}}{k_{1}!}\ldots \frac{{\bar{\lambda }}_{n}^{k}}{k_{n}!}\;=\;Z:=e^{{\bar{\lambda }}_{1}}\ldots e^{{\bar{\lambda }}_{n}} \end{array}$$

Thus, normalizing by the total, $\left\{p_{k}/Z,k\in Z_{\geq 0}^{n}\right\}$ is a probability distribution satisfying the ssCME equations. The interpretation of this solution is that at the steady state, *S*_*i*_, *i* = 1, …, *n* are *n* independent Poisson random variables with parameters ${\bar{\lambda }}_{i}$ respectively, so

$$\begin{array}{c} P\;\left[S_{1}=k_{1},S_{2}=k_{2},\ldots ,S_{n}=k_{n}\right]\;=\;e^{-({\bar{\lambda }}_{1}+\ldots +{\bar{\lambda }}_{n})}\;\frac{{\bar{\lambda }}_{1}^{k_{1}}}{k_{1}!}\frac{{\bar{\lambda }}_{2}^{k_{2}}}{k_{2}!}\ldots \frac{{\bar{\lambda }}_{n}^{k_{n}}}{k_{n}!} \end{array}$$ (17)

for *k* ≥ 0 (and zero otherwise). Next, let’s consider the case when the network satisfies additional stoichiometric constraints of the network (e.g. conservation laws):

$$\begin{array}{c} Y_{j}\;:=\;\sum_{i=1}^{n}\alpha_{ji}S_{i}=\beta_{j}\;,\;j=1,\ldots ,q\;. \end{array}$$ (18)

Observe that

PY1=β1,…,Yq=βq=∑k1,…,kn≥0α11k1+…+α1nkn=β1,…,αq1k1+…+αqnkn=βqe-(λ¯1+…+λ¯n)λ¯1k1k1!λ¯2k2k2!…λ¯nknkn!=:e-(λ¯1+…+λ¯n)Z(β1,…,βq),

where *Z*(*β*_1_, …, *β*_*q*_) is referred to as the partition function. This leads to the following conditional probability at the steady state if *k* satisfies the stochiometric constrains:

$$\begin{array}{c} P\;\left[S_{1}=k_{1},S_{2}=k_{2},\ldots ,S_{n}=k_{n}\;|\;Y_{1}=\beta_{1},\ldots ,Y_{m}=\beta_{m}\right]\;\;=\;\frac{1}{Z(\beta_{1},\ldots ,\beta_{q})}\frac{{\bar{\lambda }}_{1}^{k_{1}}}{k_{1}!}\frac{{\bar{\lambda }}_{2}^{k_{2}}}{k_{2}!}\ldots \frac{{\bar{\lambda }}_{n}^{k_{n}}}{k_{n}!} \end{array}$$

and is zero otherwise. If our interest is in computing this conditional probability, the main effort goes into computing the partition function, *Z*(*β*_1_, …, *β*_*q*_). The main contribution of the paper [62] was to provide effective algorithms for the computation of *Z*(*β*_1_, …, *β*_*q*_) recursively on the *β*_*i*_’s. A package for that purpose, called MVPoisson, was included with that paper.

Importantly, the stationary conditional moment of any species at the steady state can be obtained using the following formula:

$$\begin{array}{c} E\left[S_{j}^{r},|\;Y\right]\;\;=\;\;{\bar{\lambda }}_{j}\;\cdot \;\frac{Z(\beta_{1}-r\alpha_{1j},\beta_{2}-r\alpha_{2j},\ldots ,\beta_{q}-r\alpha_{qj})}{Z(\beta_{1},\ldots ,\beta_{q})} \end{array}$$ (19)

when all *β*_*i*_ ≥ *α*_*ij*_, and zero otherwise. Mixed moments can be also calculated using the partition function [62]. Thus, as long as the participation can be calculated, any desired stationary moments can be derived and thus model can be accurately reduced.

#### Summary of the procedure to derive stationary moments of complex balanced networks

*Step 1*. Check whether the given network is complex balanced, which can be done by checking deficiency and weak reversibility of the network.
*Step 2*. Find all conservation laws, which can be done by finding the left nullspace of the stoichiometry matrix.
*Step 3*. Choose any steady state vector $\bar{\lambda }\in R_{>0}^{n}$ of the deterministic system describing the network (usually a simple one).
*Step 4*. For the choice of $\bar{\lambda }\in R_{>0}^{n}$, derive the partition function with the stoichiometric constraints given by the conservation laws.
*Step 5*. Calculate the partition function by rewriting the sum of the partition function with minimal indices using the conservation laws, using the algorithm described in S1 Appendix. Alternatively, such calculation can be done by deriving the recursion relations among partition functions with the MVPoisson package [62].
*Step 6*. Calculate the desired stationary moments using Eq (19).

Detailed description of this procedure is illustrated in S1 Appendix using the examples of Figs 5 and 7.

## Supporting information

S1 Appendix Detailed illustration of the procedure to derive stationary moments of complex balanced networks. (PDF)
