# Supplementary material for: Reduction of multiscale stochastic biochemical reaction networks using exact moment derivation
Source: PLoS Comput Biol. 2017 Jun 5;13(6):e1005571. doi: 10.1371/journal.pcbi.1005571 (PMC5481150; doi:10.1371/journal.pcbi.1005571)
Supplement: S1 Appendix — (PDF) [file pcbi.1005571.s001.pdf]

Supplementary Information for Reduction of Multiscale Stochastic Biochemical Reaction Networks using Exact Moment Derivation

Jae Kyoung Kim[1◑*], Eduardo D. Sontag[2◑*]

1 Department of Mathematical Sciences, Korea Advanced Institute of Science and Technology, Daejeon 34131, Korea
2 Department of Mathematics and Center for Quantitative Biology, Rutgers University, New Brunswick, NJ 08903, USA

◑These authors contributed equally to this work.
* jaekkim@kaist.ac.kr or eduardo.sontag@rutgers.edu, gmail.com

Supplementary Methods

Stationary moments of a complex balanced network with a reversible binding

We illustrate how to use (19) to calculate the conditional moment of complex balanced networks with a simple example. Suppose that S_1 and S_2 can reversibly bind to form a complex S_3:

$$S_1 + S_2 \underset{\kappa_b}{\overset{\kappa_f}{\rightleftharpoons}} S_3\,. \tag{S1}$$

Since the deficiency of this network is $n_c - \ell - r = 2 - 1 - 1 = 0$ and it is reversible and hence weakly reversible as well, we know that there is a complex-balanced equilibrium and every equilibrium is complex balanced [1,2]. First, we can pick any potential steady state, $\bar{\lambda} = (1, 1, K)$, where $K := \kappa_f/\kappa_b$. Note that the choice of the steady state does not affect the final result. There are two conservations: $S_1 + S_3 = p$ and $S_2 + S_3 = n$, where p and n are determined by the initial conditions. Thus, we have $\alpha_{11} = \alpha_{13} = 1$, $\alpha_{22} = \alpha_{23} = 1$, $\alpha_{12} = \alpha_{21} = 0$, $\beta_1 = p$, $\beta_2 = n$ in (18). Due to the conservations, all p_k should vanish except those corresponding to vectors $k = (k_1, k_2, k_3)$ such that $k_1 + k_3 = p$ and $k_2 + k_3 = n$. The set consisting of all such vectors is invariant, so

$$p_k = \begin{cases} \dfrac{\bar{\lambda}_1^{k_1}}{k_1!}\dfrac{\bar{\lambda}_2^{k_2}}{k_2!}\dfrac{\bar{\lambda}_3^{k_3}}{k_3!} & \text{if } k_1 + k_3 = p \text{ and } k_2 + k_3 = n \\ 0 & \text{otherwise} \end{cases}$$

is a solution of the ssCME. In order to obtain a probability density, we must normalize them by the sum of these p_k's (i.e. the partition function, $Z(p, n)$). Because of the two conservations, the sum can be expressed in terms of just one of the indices, let us say k_1. Since $k_1 + k_3 = p$ and $k_3 \geq 0$, necessarily $k_1 \leq p$. Since $k_2 = n - k_3 = n + k_1 - p$ must be non-negative, we also have the constraint $k_1 \geq \max\{0, p - n\}$. Thus, p_k is nonzero only when $k_1 \in \{\max\{0, p - n\}, \ldots, p\}$ with $k_2 = n + k_1 - p$, $k_3 = p - k_1$, and we have:

$$Z(p, n) \;=\; \sum_{\ell=\max\{0,p-n\}}^{p} \frac{K^{p-\ell}}{\ell!\,(n + \ell - p)!\,(p - \ell)!} \;=\; \sum_{\ell=0}^{p} \frac{K^{p-\ell}}{\ell!\,n!}\binom{n}{n + \ell - p}, \tag{S2}$$

where second equality comes from the convention that $\binom{n}{n+\ell-p} := \frac{n!}{(n+\ell-p)!\,(p-\ell)!} = 0$ if $n+\ell-p<0$ or $\ell<p-n$.

In particular, we have;

$$Z(0,n) = \frac{1}{n!}\,,$$

$$Z(1,n) \;=\; \frac{Kn+1}{n!}\,,$$

$$Z(2,n) \;=\; \frac{K^2n^2+\left(-K^2+2\,K\right)n+1}{2n!}\,,$$

$$Z(3,n) \;=\; \frac{K^3n^3+\left(-3\,K^3+3\,K^2\right)n^2+\left(2\,K^3-3\,K^2+3\,K\right)n+1}{3!n!}$$

and so forth.

Another way to derive $Z(p,n)$ is using the following recursion formula, which can be obtained by using the computaional package MVPoisson from [3] based on Wilf-Zeilberger theory:

$$Z(p,n+2) \;=\; \frac{K}{n+2}\,Z(p,n) \;+\; \frac{-Kn+Kp-K+1}{n+2}\,Z(p,n+1)\,.$$

Note that by symmetry, a recursion on p can be found by exchanging n and p. Furthermore, in terms of the Gauss's hypergeometric function ${}_2\mathrm{F}_0$, we can also write:

$$Z(p,n) \;=\; \frac{{}_2\mathrm{F}_0(-n,-p;\;\;;K)}{p!\,n!}$$

Since $Z(p,n)$ is calculated, we can derive the conditional mean of the first species using (19):

$$\varphi(p,n) \;:=\; E[S_1 \,\big|\, S_1+S_3=p, S_2+S_3=n] \;=\; \frac{Z(p-1,n)}{Z(p,n)}\,. \tag{S3}$$

for $p \geq 1$, $n \geq 0$, and zero otherwise. For example,

$$\varphi(1,n) \;=\; \frac{1}{Kn+1}$$

$$\varphi(2,n) \;=\; \frac{2(Kn+1)}{K^2n^2+(-K^2+2\,K)\,n+1}\,.$$

A Matlab code allowing for the calculation of (S3) is provided in this work.

A complex balanced network with competitive reversible bindings

Here, we apply (19) to calculate stationary conditional moments of another complex balanced network, this one with two competing reversible bindings:

$$A+B \;\underset{\kappa_{b_B}}{\overset{\kappa_{f_B}}{\rightleftharpoons}}\; D \tag{S4}$$

$$A+C \;\underset{\kappa_{b_C}}{\overset{\kappa_{f_C}}{\rightleftharpoons}}\; E$$

Since the deficiency of this network is $n_c-\ell-r = 4-2-2 = 0$ and it is weakly reversible, there is a complex-balanced equilibrium and every equilibrium is complex balanced [1,2]. The steady states of the associated deterministic system satisfy $\kappa_{f_B}AB = \kappa_{b_B}D$ and $\kappa_{f_C}AC = \kappa_{b_C}E$, so one such equilibrium is

$$(1,1,1,L,K) \tag{S5}$$

where

$$L := \frac{\kappa_{f_B}}{\kappa_{b_B}}, \quad K := \frac{\kappa_{f_C}}{\kappa_{b_C}}.$$

Note that following quantities are conserved:

$$\begin{aligned} A + D + E &= n_A \\ B + D &= n_B \\ C + E &= n_C \end{aligned}$$

Subject to these constraints, we can pick the following partition function:

$$Z(n_A, n_B, n_C) = \sum_{(k_A,k_B,k_C,k_D,k_E)\in\mathcal{S}} \frac{1}{k_A!}\frac{1}{k_B!}\frac{1}{k_C!}\frac{L^{k_D}}{k_D!}\frac{K^{k_E}}{k_E!}$$

where

$$\mathcal{S} = \{(k_A, k_B, k_C, k_D, k_E) \geq 0 \,|\, k_A + k_D + k_E = n_A,\; k_B + k_D = n_B,\; k_C + k_E = n_C\}.$$

Thu sum can be re-written as a double sum because $\mathcal{S}$ is equal to the following set due to the three conservations.

$$\begin{aligned} \mathcal{S}' = \{(k_A, k_B, k_C, k_D, k_E) \quad | \quad & 0 \leq k_D \leq n_B,\; 0 \leq k_E \leq \min\{n_A - k_D, n_C\}, \\ & k_A = n_A - (k_D + k_E),\; k_B = n_B - k_D,\; k_C = n_C - k_E\}. \end{aligned}$$

Suppose that $(k_A, k_B, k_C, k_D, k_E) \in \mathcal{S}$ so that $k_i \geq 0$ for all i. Then from $k_B + k_D = n_B$, we have that $k_D = n_B - k_B \leq n_B$. Similarly, $k_C + k_E = n_C$ and $k_A + k_D + k_E = n_A$ lead to $k_E = n_C - k_C \leq n_C$ and $k_E \leq n_A - k_A - k_D \leq n_A - k_D$, respectively, so that $k_E \leq \min\{n_A - k_D, n_C\}$. Thus $(k_A, k_B, k_C, k_D, k_E) \in \mathcal{S}'$. Conversely, suppose that $(k_A, k_B, k_C, k_D, k_E) \in \mathcal{S}'$. We have that k_D and k_E are non-negative. From $k_E \leq n_A - k_D$, it follows that $k_A = n_A - (k_D + k_E) \geq 0$, from $k_D \leq n_B$, it follows $k_B = n_B - k_D \geq 0$, and from $k_E \leq n_C$, we have $k_C = n_C - k_E \geq 0$. Therefore, $(k_A, k_B, k_C, k_D, k_E) \in \mathcal{S}$. Since $\mathcal{S} = \mathcal{S}'$, $(k_A, k_B, k_C, k_D, k_E) \in \mathcal{S}$ can be described with two indices $(k_D, k_E) = (i, j)$ as $(k_A, k_B, k_C, k_D, k_E) = (n_A - i - j, n_B - i, n_C - j, i, j)$. The procedure used to find the set $\mathcal{S}'$ is a special case of an algorithmic approach described in the following section.

Using the equality $\mathcal{S} = \mathcal{S}'$, we can rewrite the partition function as follows :

$$\begin{aligned} Z(n_A, n_B, n_C) &= \sum_{i=0}^{n_B} \frac{L^i}{(n_B - i)!\, i!} \sum_{j=0}^{\min\{n_A - i, n_C\}} \frac{K^j}{((n_A - i) - j)!\,(n_C - j)!\, j!} \\ &= \sum_{i=0}^{n_B} \frac{L^i}{(n_B - i)!\, i!}\, Q(n_A - i, n_C) \\ &= \frac{1}{n_A!} \sum_{i=0}^{n_B} \binom{n_A}{i} \frac{L^i}{(n_B - i)!}\, \widetilde{Q}(n_A - i, n_C)\,, \end{aligned}$$

where we may use either alternative expressions in terms of Q or $\widetilde{Q}$ defined as follows:

$$Q(p, n) := \sum_{\ell=0}^{\min\{p,n\}} \frac{K^\ell}{(p - \ell)!(n - \ell)!\ell!}, \quad \widetilde{Q}(p, n) := p!\, Q(p, n) = \sum_{\ell=0}^{\min\{p,n\}} \binom{p}{\ell} \frac{K^\ell}{(n - \ell)!}.$$

The sum in $\widetilde{Q}$ is numerically better performed than that in Q when p is large and n is small. With a change of indices $\ell = p - \ell$, it can be shown that Q is the partition function $Z(p, n)$ given by formula (S2) for the single binding example

$$S_1 + S_2 \rightleftharpoons S_3 \,,$$

Thus, Q can also be written as

$$\frac{1}{p!\, n!}\; {}_2\mathrm{F}_0(-p, -n; \;\; ; K)\,,$$

in terms of ${}_2\mathrm{F}_0$, Gauss's hypergeometric function. When $n_B = 0$, the partition function becomes

$$Z(n_A, 0, n_C) \quad = \quad Q(n_A, n_C),$$

which is expected as when $n_B = 0$ the species B can only be zero, so the system reduces to the previous example, with $S_1 = A$, $S_2 = C$, and $S_3 = E$. When $n_B = 1$, we get

$$Z(n_A, 1, n_C) \quad = \quad Q(n_A, n_C) \,+\, L\, Q(n_A - 1, n_C).$$

Using this, the conditional mean of species D given the constraints $(n_A, 1, n_C)$ is derived by (19):

$$E[D \,|\, n_A, 1, n_C] \;=\; L\, \frac{Z(n_A - 1, 0, n_C)}{Z(n_A, 1, n_C)} \;=\; L\, \frac{Q(n_A - 1, n_C)}{Q(n_A, n_C) \,+\, L\, Q(n_A - 1, n_C)}\,.$$

Using $\widetilde{Q}$, we may write, alternatively,

$$\begin{aligned} Z(n_A, 0, n_C) \quad &= \quad \frac{1}{n_A!}\, \widetilde{Q}(n_A, n_C) \\ Z(n_A, 1, n_C) \quad &= \quad \frac{1}{n_A!} \left(\widetilde{Q}(n_A, n_C) \,+\, L\, n_A\, \widetilde{Q}(n_A - 1, n_C) \right) \end{aligned}$$

and thus, cancelling the $n_A!$ terms, and using $Z(n_A - 1, 0, n_C) = \frac{n_A}{n_A!}\, \widetilde{Q}(n_A - 1, n_C)$,

$$E[D \,|\, n_A, 1, n_C] \;=\; L\, \frac{n_A\, \widetilde{Q}(n_A - 1, n_C)}{\widetilde{Q}(n_A, n_C) \,+\, L\, n_A\, \widetilde{Q}(n_A - 1, n_C)} \tag{S6}$$

which is far better behaved numerically when n_A is large. A Matlab code allowing for the calculation of (S6) is provided in this work.

Rather than the direct summation, partition functions and thus stationary moments can also be derived using the recursion method from [3]. For this example, a third-order recursion for Z can be obtained by the algorithm MVPoisson from [3]. In order to conveniently display the recurrences, let us use the following notations. We will write Z instead of $Z(b_1, b_2, b_3)$, and a notation like $Z_i^{+\cdots+}$ means a shift of the ith argument by the indicated number of plus signs. For example, Z_3^{++} means $Z(b_1, b_2, b_3 + 2)$. There are three recurrences of order three, as follows, for each of the three arguments:

$$\begin{aligned} (3 + b_1) Z_1^{+++} \quad &= \quad LKZ \\ &- \quad (LKb_1 - LKb_2 - LKb_3 + LK - L - K) Z_1^{+} \\ &- \quad (Lb_1 - Lb_2 + Kb_1 - Kb_3 + 2\,L + 2\,K - 1) Z_1^{++} \end{aligned}$$

$$\begin{aligned} M(3 + b_3)(b_2 + 2) Z_2^{+++} \quad &= \quad (L^2 - LK) Z \\ &+ (L^2 b_1 - L^2 b_2 - LKb_1 + 2\,LKb_2 + LKb_3 - L^2 + 3\,LK + L - K) Z_2^{+} \\ &+ (LKb_1\, b_2 - LK{b_2}^2 - LKb_2\, b_3 + 2\,LKb_1 - 4\,LKb_2 - 2\,LKb_3 \\ &\qquad - 4\,LK - Lb_2 + 2\,Kb_2 - 2\,L + 4\,K) Z_2^{++} \end{aligned}$$

$$
\begin{aligned}
L(3+b_3)(b_3+2)Z_3^{+++} &= (-LK+K^2)Z \\
&+ (-LKb_1+LKb_2+2\,LKb_3+K^2b_1-K^2b_3+3\,LK-K^2-L+K)Z_3^{+} \\
&+ (LKb_1\,b_3-LKb_2\,b_3-LK{b_3}^2+2\,LKb_1-2\,LKb_2 \\
&\qquad -4\,LKb_3-4\,LK+2\,Lb_3-Kb_3+4\,L-2\,K)Z_3^{++}\,.
\end{aligned}
$$

The algorithm provides 27 initial conditions, the values of Z for the triples $(1,1,1)$, $(1,1,2)$, $(1,1,3)$, $\ldots(3,3,3)$ listed in that order. We display them as three matrices, respectively shown below. The first matrix lists the elements of the form $(1,\star,\star)$, the next one $(2,\star,\star)$, and the last one $(3,\star,\star)$. In each matrix, elements are listed in the usual matrix order: $(\star,i,j)$ is the (i,j)th entry of the matrix.

$$
\begin{bmatrix}
L+K+1 & \frac{L}{2}+K+\frac{1}{2} & \frac{L}{6}+\frac{K}{2}+\frac{1}{6} \\
L+\frac{K}{2}+\frac{1}{2} & \frac{L}{2}+\frac{K}{2}+\frac{1}{4} & \frac{L}{6}+\frac{K}{4}+\frac{1}{12} \\
\frac{L}{2}+\frac{K}{6}+\frac{1}{6} & \frac{L}{4}+\frac{K}{6}+\frac{1}{12} & \frac{L}{12}+\frac{K}{12}+\frac{1}{36}
\end{bmatrix}
$$

$$
\begin{bmatrix}
(K+1)L+K+\frac{1}{2} & (K+\frac{1}{2})L+\frac{1}{2}K^2+K+\frac{1}{4} & \kappa_1 \\
\frac{1}{2}L^2+(K+1)L+\frac{K}{2}+\frac{1}{4} & \frac{1}{4}L^2+(K+\frac{1}{2})L+\frac{1}{4}K^2+\frac{K}{2}+\frac{1}{8} & \kappa_2 \\
\frac{1}{2}L^2+\frac{1}{2}(K+1)L+\frac{K}{6}+\frac{1}{12} & \frac{1}{4}L^2+\frac{1}{2}(K+\frac{1}{2})L+\frac{1}{12}K^2+\frac{K}{6}+\frac{1}{24} & \kappa_3
\end{bmatrix}
$$

$$
\begin{bmatrix}
\frac{1}{2}(2K+1)L+\frac{K}{2}+\frac{1}{6} & \gamma_1 & \gamma_2 \\
\frac{1}{2}(K+1)L^2+\frac{1}{2}(2K+1)L+\frac{K}{4}+\frac{1}{12} & \beta_1 & \beta_2 \\
\frac{1}{6}L^3+\frac{1}{2}(K+1)L^2+\frac{1}{4}(2K+1)L+\frac{K}{12}+\frac{1}{36} & \alpha_1 & \alpha_2
\end{bmatrix}
$$

where we are using these notations:

$$
\begin{aligned}
\kappa_1 &= (\frac{K}{2}+\frac{1}{6})L+\frac{1}{2}K^2+\frac{K}{2}+\frac{1}{12} \\
\kappa_2 &= \frac{1}{12}L^2+(\frac{K}{2}+\frac{1}{6})L+\frac{1}{4}K^2+\frac{K}{4}+\frac{1}{24} \\
\kappa_3 &= \frac{1}{12}L^2+\frac{1}{2}(\frac{K}{2}+\frac{1}{6})L+\frac{1}{12}K^2+\frac{K}{12}+\frac{1}{72} \\
\gamma_1 &= \frac{1}{2}(K^2+2K+\frac{1}{2})L+\frac{1}{2}K^2+\frac{K}{2}+\frac{1}{12} \\
\gamma_2 &= \frac{1}{2}(K^2+K+\frac{1}{6})L+\frac{1}{6}K^3+\frac{1}{2}K^2+\frac{K}{4}+\frac{1}{36} \\
\beta_1 &= \frac{1}{2}(K+\frac{1}{2})L^2+\frac{1}{2}(K^2+2K+\frac{1}{2})L+\frac{1}{4}K^2+\frac{K}{4}+\frac{1}{24} \\
\beta_2 &= \frac{1}{2}(\frac{K}{2}+\frac{1}{6})L^2+\frac{1}{2}(K^2+K+\frac{1}{6})L+\frac{1}{12}K^3+\frac{1}{4}K^2+\frac{K}{8}+\frac{1}{72} \\
\alpha_1 &= \frac{1}{12}L^3+\frac{1}{2}(K+\frac{1}{2})L^2+\frac{1}{4}(K^2+2K+\frac{1}{2})L+\frac{1}{12}K^2+\frac{K}{12}+\frac{1}{72} \\
\alpha_2 &= \frac{1}{36}L^3+\frac{1}{2}(\frac{K}{2}+\frac{1}{6})L^2+\frac{1}{4}(K^2+K+\frac{1}{6})L+\frac{1}{36}K^3+\frac{1}{12}K^2+\frac{K}{24}+\frac{1}{216}
\end{aligned}
$$

so, reading-out entries from the matrices above we have, for example:

$$Z(1,1,1)=L+K+1\,,\quad Z(2,2,2)=L^2/4+(K+1/2)L+K^2/4+K/2+1/8\,,\quad Z(3,2,3)=\beta_2\,.$$

Computing reduced sums

We remark that the reduced indices for the sums defining the partition function can be obtained in a more systematic form, through the use of Smith canonical forms. Suppose

that P is a matrix in $\mathbb{Z}^{q\times n}$ that represents q conservation laws on n species. For instance,

$$P = \begin{pmatrix} 1 & 0 & 0 & 1 & 1 \\ 0 & 1 & 0 & 1 & 0 \\ 0 & 0 & 1 & 0 & 1 \end{pmatrix}$$

in the competitive binding example. We assume, as in this and other examples, that $q \leq n$ and that the matrix P has full row rank q. Under this assumption, the integer matrix P can be represented in Smith canonical form (see for example [4]), meaning that there exist two unimodular (that is to say, invertible over the ring of integers) matrices $U \in \mathbb{Z}^{q\times q}$ and $V \in \mathbb{Z}^{n\times n}$ so that

$$UPV = [\Delta\ 0]$$

where $\Delta = \text{diag}\,(\delta_1, \ldots, \delta_q)$, 0 is a $q \times (n-q)$ matrix of zeroes, and the δ_i's are the *elementary divisors* of the matrix P. The elementary divisors are unique up to sign change, there are formulas that express then in terms of the minors of P (see [4] for details). For example, for the above example, we have $U = I$ (3×3 identity matrix),

$$V = \begin{pmatrix} 1 & 0 & 0 & -1 & -1 \\ 0 & 1 & 0 & -1 & 0 \\ 0 & 0 & 1 & 0 & -1 \\ 0 & 0 & 0 & 1 & 0 \\ 0 & 0 & 0 & 0 & 1 \end{pmatrix}$$

and $\delta_1 = \delta_2 = \delta = 3 = 1$, so $UPV = [I\,0]$. In general, if we wish to find non-negative integer solutions of $Ak = b$, for a given (non-negative) integer vector b, we use that $UPVV^{-1}k = Ub$, so, using the indices $\ell = V^{-1}k$,

$$[\Delta\ 0]\ell = Ub$$

which means that the last $n - q$ indices ℓ are free, and the constraint $V\ell \geq 0$ is imposed to insure non-negativity of k. For instance, in the competitive binding example, and recalling that $U = I$ and $\Delta = I$, the equation $[\Delta\,0]\ell = Ub$ gives that $\ell_1 = b_1$, $\ell_2 = b_2$, $\ell_3 = b_3$, and $\ell_4 = i$, $\ell_5 = j$ are arbitrary. Thus we can express the sum as a sum over the two indices $k_4 = i$ and $k_5 = j$, with $k_1 = b_1 - (i + j)$, $k_2 = b_2 - i$, and $k_3 = b_3 - j$. The non-negativity condition $V\ell \geq 0$, applied with the above matrix V, says that these expressions must be non-negative: which means that the sum can be re-expressed as a sum over $i \geq 0$, $j \geq 0$, subject to $i \leq b_2$, $j \leq b_3$, and $i + j \leq b_1$. This is exactly the same as the set $\mathcal{S}'$ computed by hand.

References

1. Feinberg M. Chemical reaction network structure and the stability of complex isothermal reactors - I. The deficiency zero and deficiency one theorems. Chemical Engr Sci. 1987;42:2229–2268.

2. Feinberg M. The existence and uniqueness of steady states for a class of chemical reaction networks. Archive for Rational Mechanics and Analysis. 1995;132:311–370.

3. Sontag ED, Zeilberger D. A symbolic computation approach to a problem involving multivariate Poisson distributions. Advances in Applied Mathematics. 2010;44:359–377.

4. Jacobson N. Basic Algebra I. 2nd ed. Freeman and Company; 1995.
